# Tamoxifen treatment ameliorates contractile dysfunction of Duchenne muscular dystrophy stem cell-derived cardiomyocytes on bioengineered substrates

**DOI:** 10.1038/s41536-022-00214-x

**Published:** 2022-03-18

**Authors:** Foster Birnbaum, Asuka Eguchi, Gaspard Pardon, Alex C. Y. Chang, Helen M. Blau

**Affiliations:** 1 Baxter Laboratory for Stem Cell Biology, Department of Microbiology and Immunology, Stanford University School of Medicine, Stanford University, Stanford, CA USA; 2 Stanford Cardiovascular Institute, Stanford University School of Medicine, Stanford, CA USA; 3 Department of Cardiology and Shanghai Institute of Precision Medicine, Ninth People’s Hospital, Shanghai Jiao Tong University School of Medicine, Shanghai, China

**Keywords:** Cardiomyopathies, Induced pluripotent stem cells, Hormone receptors, Stem-cell biotechnology

## Abstract

Duchenne muscular dystrophy (DMD) is a progressive genetic myopathy that leads to heart failure from dilated cardiomyopathy by early adulthood. Recent evidence suggests that tamoxifen, a selective estrogen receptor modulator widely used to treat breast cancer, ameliorates DMD cardiomyopathy. However, the mechanism of action of 4-hydroxytamoxifen, the active metabolite of tamoxifen, on cardiomyocyte function remains unclear. To examine the effects of chronic 4-hydroxytamoxifen treatment, we used state-of-the-art human-induced pluripotent stem cell-derived cardiomyocytes (iPSC-CMs) and a bioengineered platform to model DMD. We assessed the beating rate and beating velocity of iPSC-CMs in monolayers and as single cells on micropatterns that promote a physiological cardiomyocyte morphology. We found that 4-hydroxytamoxifen treatment of DMD iPSC-CMs decreased beating rate, increased beating velocity, and ameliorated calcium-handling deficits, leading to prolonged viability. Our study highlights the utility of a bioengineered iPSC-CM platform for drug testing and underscores the potential of repurposing tamoxifen as a therapy for DMD cardiomyopathy.

## Introduction

Duchenne muscular dystrophy (DMD), a lethal X-linked recessive disease, is the most common inherited myopathy, affecting one in 5000 males^1^. DMD arises from the absence of dystrophin, a key component of the dystrophin-glycoprotein complex that anchors the cytoskeleton to the extracellular matrix and stabilizes the plasma membrane, thereby protecting the cell from contraction-induced mechanical stress^2^. More than 7100 mutations have been reported in the dystrophin gene^3^, the largest in the human genome^4^. DMD patients exhibit cell membrane damage in their skeletal and cardiac muscles, leading to a dysregulation of calcium influx^5,6^. This increase in calcium influx triggers the release of proteases and the production of reactive oxygen species (ROS), resulting in premature cardiomyocyte cell death and fibrosis^7–10^. The stiffening of the myocardium due to fibrosis leads to decreased cardiac output, exacerbating contraction-induced membrane damage, and calcium dysregulation in the remaining cardiomyocytes^6,11^. Patients typically lose the ability to walk by their teenage years, and most develop dilated cardiomyopathy, evidenced by tachycardia and decreased left ventricular function as young adults^10,12,13^. Previously, respiratory failure was the primary cause of death for DMD patients, but with advances in ventilation assist devices, cardiac arrhythmia has emerged as the leading cause of death^10,13^.

Treatment options for DMD cardiomyopathy include angiotensin-converting enzyme inhibitors, beta-adrenergic blockers, angiotensin II receptor blockers, and mineralocorticoid receptor antagonists^6,10,11,14,15^. While these drugs reduce the mechanical load on the heart, there is no cure for dilated cardiomyopathy in DMD patients, underscoring the need for the development of novel therapeutic approaches that improve cardiac function in DMD patients^15,16^. Tamoxifen, a Food and Drug Administration-approved breast cancer therapeutic agent, has been identified as a potential therapy for DMD through drug repurposing studies^17,18^. Repurposing of drugs significantly lowers the entry barrier to clinical trials because preclinical studies on toxicology have already been completed. Tamoxifen is a selective estrogen receptor modulator that acts through the estrogen receptor alpha (ERα)^19^, present in both the skeletal muscle and heart^20^. Several studies have shown estrogen hormones protect the heart from cardiovascular disease in men and premenopausal women^21,22^, and in the skeletal muscle, estrogen hormones prevent contraction-related stress^23^. In accordance, tamoxifen protects against contraction-induced membrane damage, modulates calcium influx, prevents oxidative stress, and inhibits fibrosis in various tissues, including the heart^23–26^. In a DMD mouse model, tamoxifen improved hanging times on a wire, a test of skeletal muscle function, and reduced cardiac fibrosis^18^. These findings suggest tamoxifen as a promising treatment option for DMD. Indeed, clinical trials in Israel and Switzerland are evaluating the efficacy of tamoxifen treatment in DMD patients (Clinical Trials NCT02835079, NCT03354039)^27^. However, neither clinical trial is likely to determine the specific effects of tamoxifen on the heart or its mechanism of action because the outcome measures of these trials are designed to assess efficacy only in the skeletal muscles. In addition, the onset of cardiac symptoms in patients is variable and delayed with respect to skeletal muscle degeneration, and the progression leading to DMD cardiomyopathy spans a time frame beyond the length of these clinical trials^6,10^. Therefore, an analysis of the effects of tamoxifen in human DMD cardiomyocytes is timely and would inform our understanding of the potential of this drug to delay the onset of dilated cardiomyopathy.

As we and others have shown, human-induced pluripotent stem cell-derived cardiomyocytes (iPSC-CMs) are a useful in vitro disease model that enables drug testing^28–31^. Indeed, iPSC-CMs with DMD mutations exhibit dystrophin deficiency, aberrant calcium handling, and impaired contractile function^31^. Although iPSC-CMs enable drug testing in human cells, one major drawback of using iPSC-CMs as a disease model is that they remain phenotypically immature^32–34^. The maturity of iPSC-CMs can affect their response to drugs, including those that modulate contractile function^35^. However, biophysical cues from the microenvironment are important determinants of cell fate^36^, and bioengineering advances now provide a means to improve the contractile and structural maturity of iPSC-CMs^37^. Notably, we and others have shown that micropatterning induces iPSC-CMs to adopt a more physiologically relevant morphology with elongated length to width aspect ratios and parallel myofibrils^31,38^, which improves the contractile function of these cells^39^. The contractile function of iPSC-CMs can be quantified using live-cell, high-throughput video analysis that applies optical flow algorithms to measure beating rate and to calculate maximum beating velocity^40^. This type of analysis has enabled the measurement of contractile dynamics in response to beta-adrenergic agonists and antagonists as well as ion channel blockers^40,41^.

In this study, we assess the effects of 4-hydroxytamoxifen, the active metabolite of tamoxifen, on the contractile function of healthy and DMD iPSC-CMs. These cardiomyocytes express estrogen receptor alpha (ERα), making them a suitable model for testing tamoxifen and its metabolites^42^. By culturing human iPSC-CMs as monolayers, the effects of cell–cell interactions can be assessed. By using a bioengineered platform with a single iPSC-CMs on a micropatterned extracellular matrix, cell-intrinsic responses to drugs can be evaluated in the context of a more mature phenotype with myofibrils aligned in parallel. Here, we test chronic 4-hydroxytamoxifen treatments in monolayers and as single cells. Our data suggest that the therapeutic effects of 4-hydroxytamoxifen on DMD cardiomyocytes arise from enhancements of contractile function, calcium handling, and viability.

## Results

### Validation of the iPSC-CM platform

Modeling cardiovascular disease using iPSC-CMs harboring patient mutations has underscored their utility for drug testing^28–31^. We used two pairs of healthy and DMD iPSC lines: one line (Healthy1) was derived from a healthy patient, and the isogenic DMD line was CRISPR-induced to yield the mutation c.263delG (DMD1)^43,44^. The second DMD line was derived from a patient with the mutation c.4918_4919AC > TG (DMD2), and the isogenic healthy line was CRISPR-corrected to eliminate the mutation (Healthy2)^45,46^. Accordingly, each DMD line was compared to a corresponding healthy control line in the same genetic background. Pluripotency of healthy and DMD iPSCs was confirmed by immunostaining with the classic stem cell markers, OCT4, SOX2, and TRA-1-60 (Supplementary Fig. 1).

**Fig. 1:**
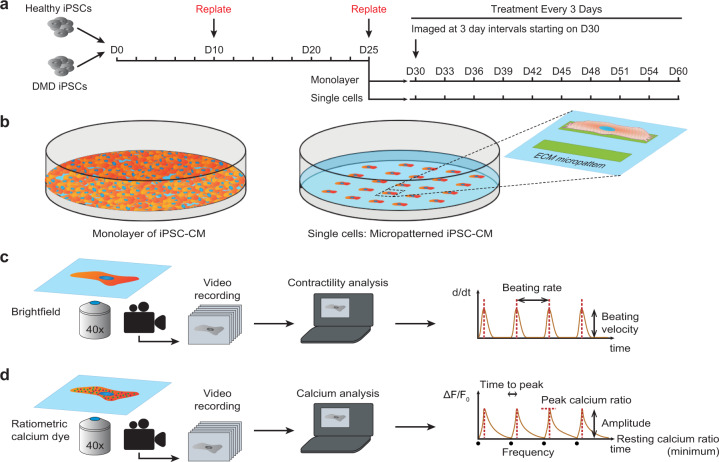
Experimental design for chronic 4-hydroxytamoxifen treatment. **a** Cells are replated on day 10 (D10), then seeded onto our bioengineered platform on day 25 (D25). Cells are imaged and treated every 3 days from day 30 (D30). **b** Schematic of iPSC-CMs cultured in a monolayer (left) and as single cells on extracellular matrix (ECM) micropatterns with a length to width aspect ratio of 7:1 (right). **c** Motion-tracking analysis was used to measure the beating rate and beating velocity of the iPSC-CM monolayers and single cells. Videos of contracting cardiomyocytes are recorded, and analysis is performed to calculate speed of contraction (d/d*t*). The beating rate is defined as the number of contraction cycles per unit of time. The beating velocity is defined as the maximum contraction speed. **d** Ratiometric analysis of calcium flux in iPSC-CM single cells. Videos of contracting cardiomyocytes loaded with a ratiometric calcium dye are recorded, and analysis is performed to measure calcium bound dye and calcium-free dye relative to baseline (Δ*F*/*F*_0_). Time to peak is defined as the time between the local minimum at the start of the peak and the next local maximum. Transient amplitude is defined as the difference between the maximum Δ*F/F*_0_ and the minimum Δ*F/F*_0_ for each peak. Frequency is the number of calcium oscillations per second. The resting calcium ratio is the minimum Δ*F/F*_0_. Peak calcium ratio is the maximum Δ*F/F*_0_.

To test the effects of 4-hydroxytamoxifen, we cultured iPSC-CMs in monolayers and on micropatterns (Fig. 1a, b). The monolayers are advantageous as they provide the iPSC-CMs with cell–cell interactions. On the other hand, the single cells seeded on micropatterns enable cell-intrinsic contractility measurements with greater precision. We evaluated the effects of chronic treatment of 4-hydroxytamoxifen on DMD iPSC-CMs starting on day 30 of differentiation. For both the monolayers and single cells, we treated the cells every 3 days and used live-cell video analysis to measure beating rate and maximum beating velocity (Fig. 1c). In addition, the single-cell platform enabled measurements of calcium flux using the ratiometric calcium dye, Fura Red, which reveals individual cell responses to pacing that cannot be captured from cells cultured in monolayers (Fig. 1d).

**Fig. 2:**
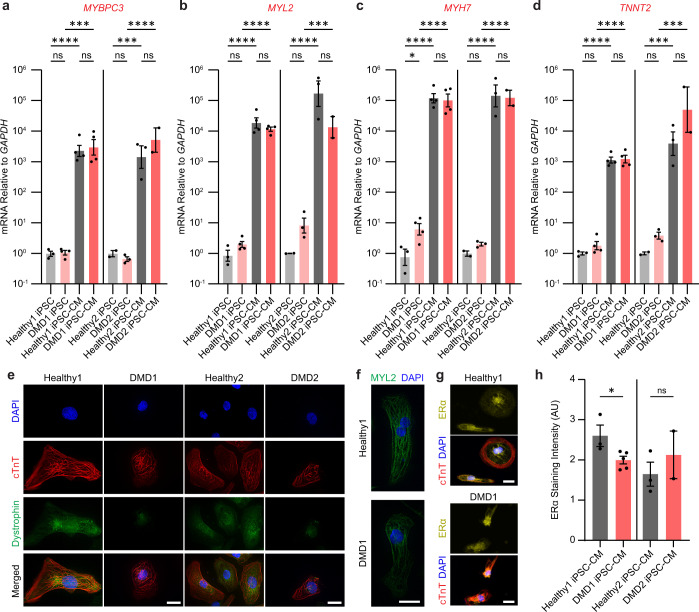
Characterization of iPSC-CMs. **a**–**d** RT-qPCR results from iPSCs and iPSC-CMs of cardiac lineage markers, *MYBPC3*, *MYL2*, *MYH7*, and *TNNT2*. Data represent mean ± SEM. Statistical significance determined by one-way ANOVA and Tukey test for post hoc comparison. *P < 0.05, **P < 0.01, ***P < 0.001, ****P < 0.0001, ns (nonsignificant). *n* = 2–4 biological replicates. **e** 3D deconvoluted micrographs of iPSC-CMs stained for DAPI (blue), cTnT (red), and dystrophin (green) taken at ×63 magnification. Healthy1 and DMD1 iPSC-CMs were stained with MANEX1A. Healthy2 and DMD2 iPSC-CMs were stained with ab15277. Detection of the short Dp71 isoform of dystrophin likely accounts for the nuclear staining. **f** 3D deconvoluted micrographs of iPSC-CMs stained with DAPI (blue) and MYL2 (green) taken at ×63 magnification. **g** Micrograph of iPSC-CMs stained with ERα (yellow), cTnT (red), and DAPI (blue) immediately after treatment with 4-hydroxytamoxifen taken at ×40 magnification. Scale bars represent 20 μm. **h** Intensity of ERα staining over cell area in arbitrary units. Data represent mean ± SEM. Statistical significance determined by unpaired *t* test. *P < 0.05, ns (nonsignificant). *n* = 2–5 biological replicates. *N* = 2818–9996 cells.

We validated that hallmarks of cardiac differentiation (*MYBPC3*, *MYL2*, *MYH7*, *TNNT2, TPM1*, and the adult isoform of *TNNT2*) were expressed on day 30 (Fig. 2a–d and Supplementary Fig. 2a, b). Immunostaining for cardiac troponin T (cTnT) and myosin light chain 2 (MYL2) confirmed that cardiac-specific markers were present at the protein level (Fig. 2e, f and Supplementary Fig. 2c, d). For dystrophin, we confirmed that Healthy1 and Healthy2 iPSC-CMs express dystrophin along the cytoskeleton by day 30 of differentiation (Fig. 2e). For the DMD lines, we used two different antibodies to confirm that they have the expected mutations and absence of full-length dystrophin. Use of the N-terminal antibody (MANEX1A) revealed no cytoplasmic dystrophin signal in the DMD1 iPSC-CMs (Fig. 2e). As previously characterized by western blot and mass spectrometry, the mutation in DMD1 results in the expression of a truncated dystrophin missing the N-terminal actin-binding domain encoded by exons 1–6^44^. Immunostaining with a C-terminal antibody (ab15277) demonstrated that this truncated variant, which is missing the essential actin-binding domain, is present in DMD1 iPSC-CMs (Supplementary Fig. 2e). Notably, this partial dystrophin protein is associated with early onset dilated cardiomyopathy^47^. DMD2 harbors a nonsense mutation that results in a dystrophin null phenotype as previously measured by western blot^46^ and here by immunostaining with the C-terminal antibody, confirming the lack of cytoplasmic dystrophin expression in DMD2 iPSC-CMs (Fig. 2e). The nuclear signal that is detected in the DMD lines is the Dp71 isoform, the short isoform of dystrophin that is ubiquitously expressed^46,48,49^. These studies validated that our iPSC-CM model system for the two DMD and Healthy samples have the appropriate molecular context to test drug efficacy. Beta-dystroglycan, a component of the dystroglycan complex that anchors dystrophin to the extracellular matrix^50^, was detected in iPSC-CMs regardless of mutational status, suggesting that the expression levels of the dystroglycan complex is not dependent on dystrophin levels (Supplementary Fig. 2f, g)^51^. Importantly, immunostaining for the nuclear receptor, ERα, revealed that both lines of Healthy and DMD iPSC-CMs express the receptor that binds 4-hydroxytamoxifen at nearly equivalent levels after treatment, indicating that this system is suitable for testing this drug (Fig. 2g, h). Localization of ERα was both nuclear and cytoplasmic with similar protein levels in each compartment as expected (Supplementary Fig. 2h)^52,53^. Notably, treatment with 4-hydroxytamoxifen does not affect the translocation of ERα to the nucleus; instead, this competitive inhibitor prevents activation of estrogen-responsive genes by inducing a conformational change in the receptor dimer that prevents it from binding coactivators necessary for transcription^54^.

**Fig. 3:**
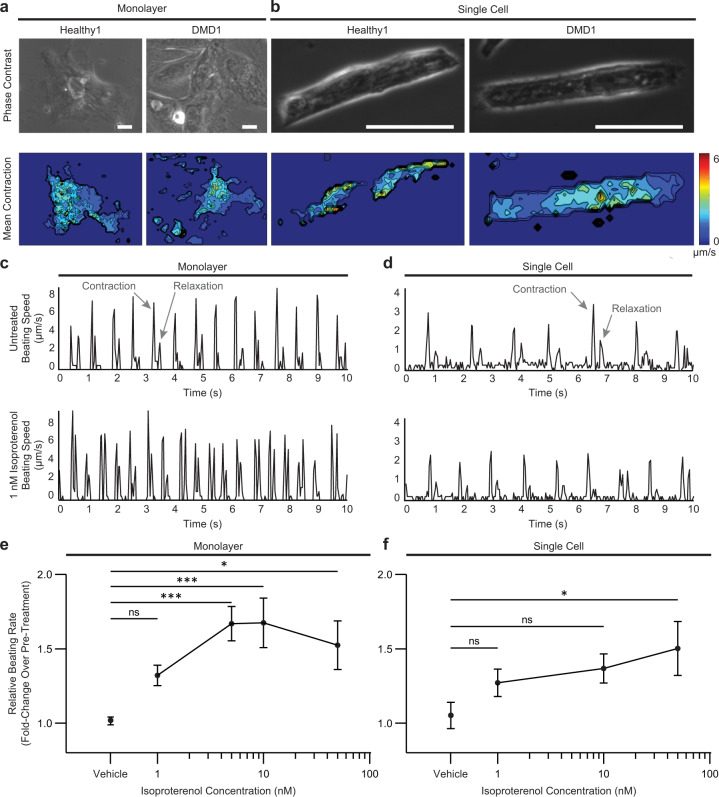
Video analysis software captures responses to pharmacological stimulation. **a**, **b** Representative phase contrast (top) and contractile heatmaps (bottom) for iPSC-CMs in monolayers and as single cells. Scale bars represent 25 μm. **c**, **d** Representative traces of speed over time at baseline (top) and after treatment with 1 nM isoproterenol (bottom) in Healthy1 iPSC-CMs cultured in monolayers and as single cells. Beating speed traces show peaks for both the contraction and relaxation cycles of contraction. Examples of peaks depicting contraction and relaxation are indicated by arrows. **e**, **f** Relative beating rate represented as fold change over pre-treatment of Healthy1 iPSC-CMs cultured in monolayers and as single cells in response to increasing concentrations of isoproterenol. Data represent mean ± SEM. Statistical significance determined by one-way ANOVA and Tukey test for post hoc comparison. *P < 0.05, ***P < 0.001, ns (nonsignificant). *n* = 3 biological replicates. *N* = 6–17 regions for monolayers and *N* = 8–24 cells for single cells.

We used an established live-cell contractility analysis algorithm^40^ to image spontaneous contractions using our bioengineered platform in healthy and DMD iPSC-CMs cultured in monolayers and as single cells (Fig. 3a, b). The contractile heatmaps accurately identified the regions of highest beating motion, and these regions of high beating motion were detected spatially with better resolution in single cells than in the monolayers (Fig. 3a, b). To validate our bioengineered platform, we measured the contractile dynamics of Healthy1 iPSC-CMs in response to isoproterenol, a well-characterized beta-adrenergic receptor agonist^40,41,55,56^. We tested a range of concentrations that encompass the free plasma concentrations detected during clinical use (effective therapeutic plasma concentration of 1.75 nM)^41^. From the contractility analysis algorithm^40,41^, we acquired traces of beating speed that show peaks at the contraction and relaxation cycles of contraction (Fig. 3c, d). Representative beating speed traces showed an increase in beating rate (i.e., the number of contraction peaks per second) in response to isoproterenol, confirming that this assay can accurately detect a response to a drug that increases the heart rate (Fig. 3c, d). Further, the magnitude of the contraction peak was used to calculate beating velocity. Upon treatment with isoproterenol, the beating rate increased, and this response was dose-dependent (Fig. 3e, f), consistent with a previous report showing a similar dose response when electrophysiological measurements were made with microelectrode arrays^57^. We found that the cells cultured in monolayers were more sensitive to lower concentrations of isoproterenol than single cells (Fig. 3e, f). On the other hand, individual cells micropatterned to promote cardiomyocyte maturation through the parallel alignment of myofibrils exhibited a progressive response to increasing concentrations of isoproterenol (Fig. 3e, f). When cultured in monolayers, there is an all-or-none response to isoproterenol due to cell–cell interactions and the electrical coupling of the connected cardiomyocytes; in contrast, an extended dose response is observed in the micropatterned single cells in which cell-intrinsic properties are measured. Taken together, the expression of cardiomyocyte markers in iPSC-CMs and the results of the contractility assay in response to isoproterenol validates our bioengineered platform for drug testing.

### Treatment with 4-hydroxytamoxifen reduces beating rate and prolongs survival on the monolayer platform

**Fig. 4:**
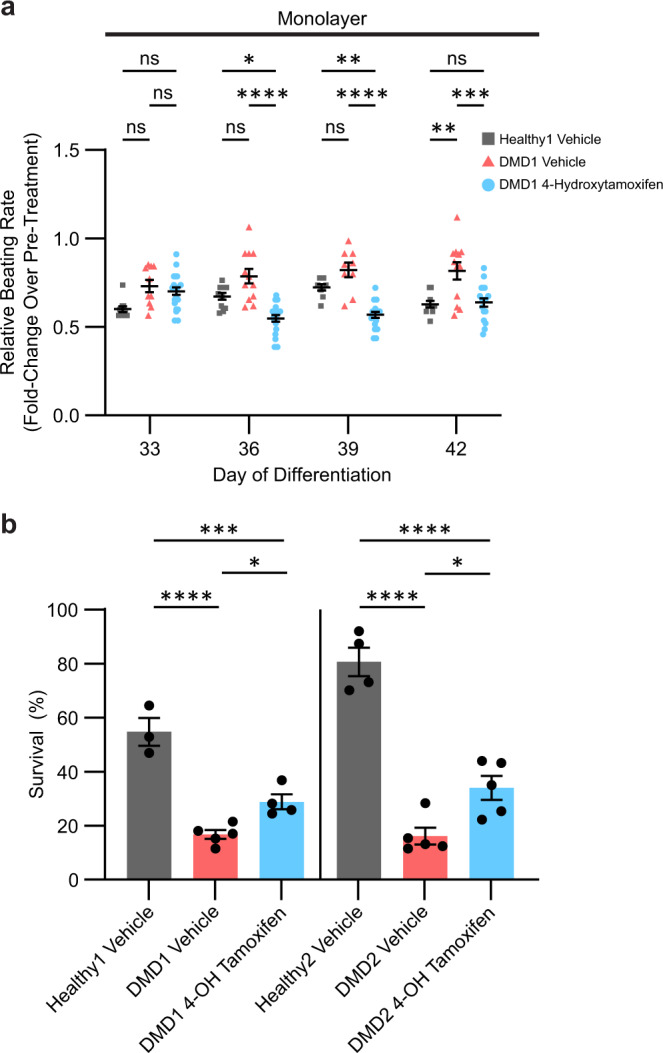
Treatment with 4-hydroxytamoxifen decreases the beating rate and improves cell survival in monolayers. **a** Relative beating rate represented as fold change over pre-treatment (day 30) to chronic 0.5 μM 4-hydroxytamoxifen or vehicle. *n* = 3 biological replicates, *N* = 9–22 regions. **b** Cell survival determined by Calcein Blue AM positive area on day 42 of differentiation relative to pre-treatment (day 30). *n* = 3–5 biological replicates. Cells were treated every 3 days beginning on day 30. Data represent mean ± SEM. Statistical significance determined by one-way ANOVA and Tukey test for post hoc comparison. *P < 0.05, **P < 0.01, ***P < 0.001, ****P < 0.0001, ns (nonsignificant).

The aim of current heart medications prescribed to DMD patients is to slow the beating rate in order to reduce the mechanical load on the dysfunctional heart^7,10^. To assess the effects of 4-hydroxytamoxifen on cardiomyocyte contractile dynamics, we tested a 30-day treatment in iPSC-CMs in monolayers starting on day 30 of differentiation. Beating rates of spontaneous contractions were normalized to day 30 of differentiation before treatment as previously described^40^. When cultured in monolayers, the beating rate significantly decreased in DMD1 iPSC-CMs on day 36 of differentiation, and this effect was maintained over time, which suggests tamoxifen will have the desired beneficial effect on cardiac burden or mechanical load (Fig. 4a). Furthermore, these data indicate that the cardiomyocytes remained sensitive to 4-hydroxytamoxifen treatment after repeated dosing every three days, suggesting that a repeated dosing regimen does not result in desensitization and could be applicable to patients. We observed no effect on beating velocity with 4-hydroxytamoxifen treatment in DMD1 iPSC-CMs (Supplementary Fig. 4b). Beyond day 45 of differentiation, the viability of DMD1 iPSC-CMs had declined, precluding analyses of contractility (Supplementary Figs. 3a, b and 4a, b). By contrast, Healthy1 iPSC-CMs maintained their beating capacity well into day 60 of differentiation (Supplementary Figs. 3a, b and 4a, b). By slowing the beating rate, 4-hydroxytamoxifen shows promise as a potential therapeutic to delay the onset of dilated cardiomyopathy.

To assay for cell viability, we imaged Calcein Blue AM, a cell-permeable dye that becomes fluorescent when cleaved by intracellular esterases. Treatment with 4-hydroxytamoxifen significantly increased survival of DMD1 iPSC-CMs from 17 ± 2% with vehicle treatment to 29 ± 6% with 4-hydroxytamoxifen treatment (Fig. 4b). For DMD2 iPSC-CMs, the percentage of cell survival also increased significantly from 16 ± 3% with vehicle treatment to 34 ± 5% with 4-hydroxytamoxifen treatment. Thus, the data from both lines demonstrate that 4-hydroxytamoxifen treatment prolongs cell survival, underscoring the potential for tamoxifen in preventing the premature loss of cardiomyocytes in the DMD heart.

### Treatment with 4-hydroxytamoxifen prolongs beating capacity, reduces beating rate, and increases beating velocity in micropatterned single cells

**Fig. 5:**
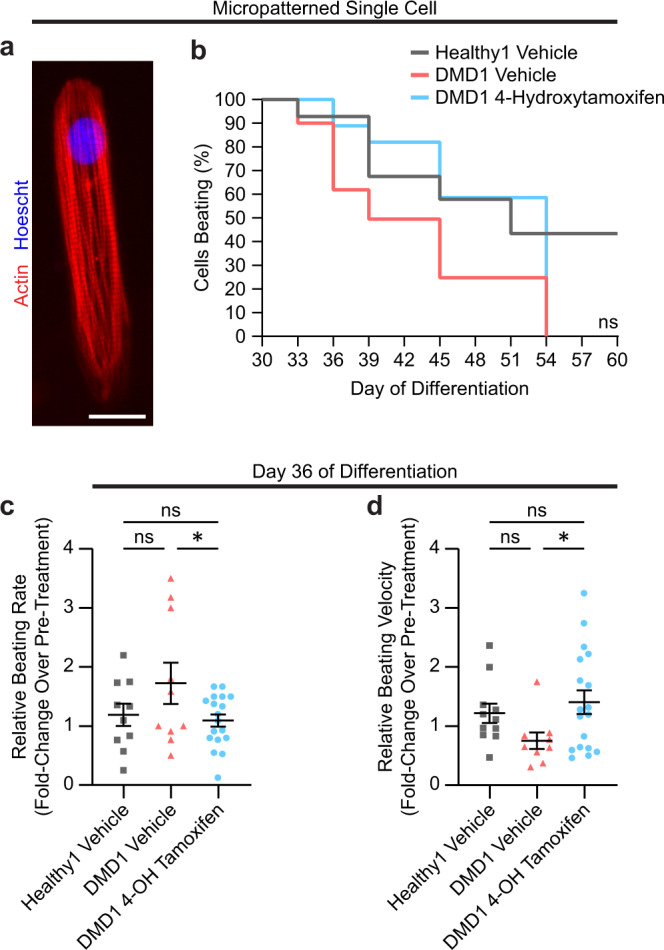
Treatment with 4-hydroxytamoxifen prolongs cell survival, decreases beating rate, and increases beating velocity in micropatterned single cells. **a** Micrograph of a patterned iPSC-CM stained with F-actin (red) and Hoescht (blue) taken at ×40 magnification. Scale bar represents 20 μm. **b** Percentage of iPSC-CMs that retained the ability to spontaneous beat as individual cells. *n* = 3 biological replicates, *N* = 14–29 cells. Statistical significance determined by log-rank (Mantel–Cox) test with Bonferroni correction. **c**, **d** Relative beating rate and relative beating velocity on day 36 of differentiation represented as fold change over pre-treatment (day 30) to chronic 0.5 μM 4-hydroxytamoxifen or vehicle. Cells were treated every 3 days beginning on Day 30. Data represent mean ± SEM. Statistical significance determined by one-way ANOVA and Tukey test for post hoc comparison. *P < 0.05, ns (nonsignificant). *n* = 3 biological replicates, *N* = 9–18 cells.

In parallel with the monolayer experiments, we performed a 30-day treatment of single cells on a micropatterned extracellular matrix that fosters a physiological shape with aligned myofibrils, which promotes the maturation of iPSC-CMs (Fig. 5a)^38^. Cells were replated on micropatterns as single cells on day 25 of differentiation, and treatments were initiated on day 30. The percentage of cells that continued to spontaneously beat was assessed from day 30 to day 60 of differentiation. Treatment with 4-hydroxytamoxifen increased the percentage of cells that retained beating capacity on micropatterns by 34% in DMD1 iPSC-CMs on day 51 (Fig. 5b) in agreement with the 12–18% increased cell viability observed with monolayers (Fig. 4b). The viability of DMD1 iPSC-CMs was severely compromised by day 39 of differentiation (Supplementary Fig. 5a, b). Even Healthy1 iPSC-CMs did not survive beyond day 48 of differentiation when micropatterned as single cells, highlighting the importance of cell–cell interactions in long-term viability (Supplementary Fig. 5a, b).

Individual cells were tracked over a 30-day treatment period for the analysis of spontaneous contractions. The beating rate for DMD1 iPSC-CMs was higher than that for vehicle-treated Healthy1 iPSC-CMs but decreased with 4-hydroxytamoxifen treatment, consistent with the results obtained in the monolayer experiments (Fig. 5c). This finding suggests that 4-hydroxytamoxifen reduces the beating rate in two independent assays. In the micropatterned single cells, the magnitude of the beating speed signal-to-noise ratio was markedly greater than in the monolayers, enabling the beating velocity to be assessed. The beating velocity increased in DMD1 iPSC-CMs on day 36 of differentiation and was on par with Healthy1 iPSC-CMs (Fig. 5d). Previously, we reported that DMD iPSC-CMs exhibit reduced contraction velocity compared to healthy controls on substrates that mimic a fibrotic myocardium^31^. The increase in beating velocity we observe here suggests that treatment with 4-hydroxytamoxifen improves the contractile function of DMD iPSC-CMs. Assessment of micropatterned single cells revealed that 4-hydroxytamoxifen prolongs beating capacity, decreases beating rate, and increases beating velocity in DMD iPSC-CMs. The single-cell experiments reinforce the findings in the monolayer experiments, demonstrating that tamoxifen has the potential to delay the loss of cardiomyocytes in the heart and slow the heart rate to delay the onset of dilated cardiomyopathy. Moreover, the single-cell experiments enable the assessment of beating velocity due to the improved signal-to-noise ratio compared to the monolayers. The results reveal that beating velocity, which is compromised in DMD iPSC-CMs^31^, is markedly improved with 4-hydroxytamoxifen treatment, suggesting that tamoxifen treatment in DMD patients holds promise in restoring contractile function in diseased cardiomyocytes.

### Treatment with 4-hydroxytamoxifen reduces arrhythmic calcium-handling events and lowers the resting calcium ratio

**Fig. 6:**
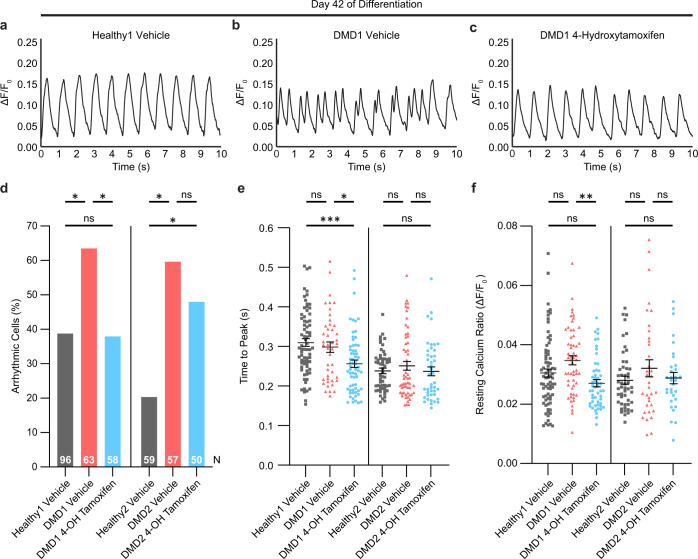
Treatment with 4-hydroxytamoxifen reduces the percentage of arrhythmic cells and lowers resting calcium ratio. **a**–**c** Representative calcium transients in iPSC-CMs on day 42 of differentiation. Calcium imaging was performed after cells were treated every 3 days beginning on day 30. **d** Percentage of cells exhibiting arrhythmic events on day 42. Pearson’s chi-square test was used to compare frequencies of arrhythmic cells. Z-test with Bonferroni correction was used for pairwise comparisons. *n* = 3 biological replicates, N indicates total number of cells analyzed. *P < 0.05. **e**, **f** Time to peak and resting calcium ratio on day 42. Cells were treated every 3 days beginning on day 30 of differentiation. Data represent mean ± SEM. Statistical significance determined by one-way ANOVA and Tukey test for post hoc comparison. *P < 0.05, **P < 0.01, ns (nonsignificant). *n* = 3 biological replicates, *N* = 32–75 cells.

Since elevated calcium levels are a consistent characteristic of DMD cardiomyocytes due to the leaky plasma membrane^4,6^, we investigated whether 4-hydroxytamoxifen treatment could ameliorate calcium-handling deficits and arrhythmic events. For this purpose, the single-cell platform is ideal as it enables the cells to be paced by electrical stimulation to contract at regular intervals. Previously, we and others have shown that DMD iPSC-CMs exhibit aberrant calcium handling^31,44,51^. Here we investigated the effect of 4-hydroxytamoxifen on calcium transients in single micropatterned iPSC-CMs paced at 1 Hz. Unlike spontaneous contractions that can be quite heterogenous among iPSC-CMs that are genetically identical, pacing provides calcium flux data in response to electrical stimulation at regular intervals, reducing variability in the results. Representative calcium traces demonstrate that vehicle-treated Healthy1 iPSC-CMs exhibit regular calcium oscillations that peak at 1 s intervals (Fig. 6a). In DMD1 iPSC-CMs, calcium peaks were characterized by early afterdepolarization events marked by double-humped peaks, indicative of calcium spikes during the decay phase of the calcium transients. Treatment with 4-hydroxytamoxifen treatment ameliorated these early afterdepolarization events in DMD1 iPSC-CMs on day 42 of differentiation (Fig. 6b, c). Because early afterdepolarization events can cause lethal cardiac arrhythmias^58^, correction of such events would be beneficial to the DMD heart.

Comprehensive analysis of calcium transients showed that 4-hydroxytamoxifen treatment significantly reduced the percentage of cells exhibiting arrhythmic traces in DMD1 iPSC-CMs (Fig. 6d). In Healthy1 iPSC-CMs, 39% of vehicle-treated cells exhibited arrhythmic traces. Treatment with 4-hydroxytamoxifen significantly reduced the percentage of arrhythmic DMD1 iPSC-CMs from 64 to 38% on par with Healthy1 iPSC-CMs (Fig. 6d). Rescue by 4-hydroxytamoxifen was also observed in the second isogenic pair. In Healthy2 iPSC-CMs, 20.3% of vehicle-treated cells exhibited arrhythmic traces, and we observed a reduction in the percentage of arrhythmic cells from 60 to 48% in DMD2 iPSC-CMs with 4-hydroxytamoxifen treatment (Fig. 6d). The peak frequency, peak calcium ratio, and transient amplitude did not change significantly in response to drug treatment (Supplementary Fig. 7a–c). Importantly, key parameters of calcium handling were altered. For example, time to peak was significantly reduced in DMD1 iPSC-CMs upon treatment with 4-hydroxytamoxifen (Fig. 6e). This faster time to peak is consistent with the increase in beating velocity we observe in the contractility analysis. In addition, the resting calcium ratio in DMD1 iPSC-CMs was significantly reduced to levels comparable to Healthy1 iPSC-CMs (Fig. 6f). Since elevated calcium levels are a hallmark of DMD cardiomyocytes, the reduction of the resting calcium ratio with drug treatment suggests that tamoxifen can have a beneficial effect on the DMD heart. In summary, single-cell analysis of calcium handling shows that 4-hydroxytamoxifen ameliorates early afterdepolarization events, reduces the frequency of arrhythmic events, and lowers the resting calcium ratio. These calcium results provide insights into the mechanism by which tamoxifen can prevent lethal arrhythmias.

## Discussion

Cardiomyopathy is the leading cause of mortality in DMD patients. Thus, therapies that slow the onset of heart failure are of major interest and an active area of investigation. Here, we show that 4-hydroxytamoxifen ameliorates cellular dysfunction exhibited by DMD cardiomyocytes differentiated from patient-derived iPSCs, demonstrating the efficacy of this drug in the molecular context of human diseased cells. We use two isogenic pairs of iPSC-CMs as disease models, which enables the effects of the two dystrophin mutations to be directly compared to normal dystrophin in cardiomyocytes with the same genetic background, underscoring the robustness of our findings. Further, we were able to compare cardiomyocytes on two different platforms. The culture of the cardiomyocytes as monolayers enabled us to evaluate the effects of the drug in a context in which cell–cell interactions are maintained. To evaluate cell-intrinsic differences, we used our bioengineered platform to micropattern single iPSC-CMs to a 7:1 length:width aspect ratio that enhances myofibril alignment and improves the maturity of these cells^37,38^. Using these two complementary approaches, we provide fresh insights into the effects of 4-hydroxytamoxifen on cardiomyocyte function. We demonstrate that 4-hydroxytamoxifen reduces the beating rate of DMD iPSC-CMs, suggesting that tamoxifen can ameliorate tachycardia in the DMD heart. Additionally, we show that the effects of 4-hydroxytamoxifen are maintained over a 12-day period, which reveals that desensitization after repeated dosing does not occur. Since DMD patients in the clinical trials receive a daily dose of tamoxifen^27^, it is important that ERα does not become unresponsive with repeated exposure to the ligand.

Our contractility assays revealed that the spatial and temporal resolution of localized motion is greater in micropatterned single cells compared to cells cultured in a monolayer, improving the signal-to-noise ratio and the sensitivity of the beating velocity measurement. This difference is due in part to the physiological shape fostered by micropatterning that promotes alignment of the myofibrils and enhances mechanical contraction. The capacity to acquire the beating velocity measurement does not reflect differences in gene expression profiles of micropatterned cells and unpatterned cells, as a previous report showed that cardiac markers are not significantly altered in cardiomyocytes cultured on the two platforms^38^. Due to spatial resolution, measurements of beating velocity in single cells were obtained with the bioengineered platform that were precluded by the cell density in monolayers. Our data show that beating velocity increases with 4-hydroxytamoxifen treatment, demonstrating an improvement in contractile function. While micropatterned cells provide better systems to measure both beating rate and beating velocity, we found that iPSC-CMs cultured in monolayers are more useful systems to test the effects of drugs over a longer period of time. Cell–cell contacts are important for maintaining long-term cultures. Monolayer cultures allowed us to robustly measure the chronic effect of 4-hydroxytamoxifen on beating rate which was not possible in isolated single cells, which underwent cell death in a shorter time frame. Notably, in cells cultured in monolayers and as micropatterned single cells, treatment with 4-hydroxytamoxifen prolonged cell survival and beating capacity, respectively, highlighting the mechanisms by which tamoxifen administration could prevent the loss of cardiomyocytes in the DMD heart. We observed different responses between the two pairs of lines to 4-hydroxytamoxifen treatment with respect to calcium handling, possibly due to the expression of truncated dystrophin in DMD1 and the complete lack of dystrophin expression in DMD2. Notably, the finding that 4-hydroxytamoxifen rescues arrhythmic calcium transients and lowers the resting calcium ratio suggests that this mechanism may underly the drug’s effects in modulating contractile function and cell viability.

Our observation that tamoxifen prolongs the survival of cardiomyocytes suggests that treatment could delay cardiomyocyte loss and subsequent fibrosis in the DMD heart. Delaying premature cardiomyocyte loss is particularly important in the context of increased tissue stiffness, as we have previously shown that DMD iPSC-CMs exhibit reduced force of contraction on stiff substrates mimicking a fibrotic myocardium compared to healthy isogenic controls^31^. By delaying cell death and improving contractile function, 4-hydroxytamoxifen could slow the cycle of contraction-mediated ROS production, activation of the DNA damage response, loss of cardiomyocytes, fibrosis, and increased tissue stiffening^6–10,59^. In *mdx* mice, tamoxifen has been shown to reduce fibrosis in the heart^18^; however, rescue of heart function by echocardiography was not measured. Indeed, *mdx* mice have a mild cardiac phenotype, and surprisingly, dilated cardiomyopathy does not manifest in males despite this disease being X-linked^60^. Thus, it is difficult to study the efficacy of drugs ameliorating cardiac function in the *mdx* model. Instead, studies in animal models of DMD that exhibit dilated cardiomyopathy, such as our mdx^4*cv*^/mTR^G2^ mice^8,61^, may provide more insight on whether tamoxifen can preserve ejection fraction and delay the onset of heart failure.

For decades, tamoxifen has been a widely used drug to treat breast cancer^62^. As a nonsteroidal competitive inhibitor of estradiol, the predominant form of estrogen in men and premenopausal women^63,64^, tamoxifen prevents activation of estrogen-responsive gene promoters^65^. When the hormone, estradiol, binds ERα, the receptor forms a dimer and translocates to the nucleus to bind estrogen response elements in the genome, inducing cell proliferation in the context of cancer^54^. By contrast, binding of tamoxifen to ERα still allows dimerization and translocation to the nucleus but does not induce the conformational change necessary for coactivators to be recruited^66,67^. Estradiol can also bind G protein-coupled estrogen receptor 1 (GPER1) and induce more rapid protein kinase-mediated signaling^68^. Similarly, tamoxifen and its metabolites can function as agonists of GPER1^69^. A report on guinea pig cardiomyocytes demonstrated that estradiol decreases the beating rate as well as cell shortening^70^ but whether this effect is dependent on GPER1 remains to be elucidated. Breast cancer patients treated with tamoxifen showed a reduced risk of coronary heart disease^71^, motivating further studies on the cardiovascular benefits of tamoxifen, including lowering cholesterol levels^72–75^. Our results show that tamoxifen is beneficial to the heart by ameliorating cardiomyocyte contractile function. Thus, the effects of tamoxifen on cardiovascular health appear to be multifaceted.

In summary, our findings highlight that repurposing of the breast cancer drug, tamoxifen, may be beneficial to DMD patients with cardiomyopathy, as the active metabolite improves contractile function and delays premature cell death in DMD cardiomyocytes. A comparison of results from monolayers and our bioengineered single-cell platform shows that performing assays in both contexts can provide an in-depth examination of how small molecules can affect cardiomyocyte function, establishing a new approach to study drug efficacy. These findings underscore the utility of performing a clinical trial in a dish.

## Methods

### iPSC-CM differentiation

The iPSC lines were gifts from David L. Mack, Martin K. Childers, and Chris Denning. DMD1 line (UC1015.6) was generated from the patient-derived healthy line, Healthy1 (UC3.4), by a CRISPR-induced mutation^43,44^. The DMD2 line (DMD19) was derived from a patient and the healthy line, Healthy2 (DMD19 iso) was generated by CRISPR-correction of the dystrophin mutation^45,46^. Both lines were differentiated into cardiomyocytes using an established protocol^76–78^. Pluripotent cells were maintained in Nutristem media (Reprocell 01-0005) on Matrigel (Corning 356231)-coated plates with daily media change. iPSCs were passaged with Accutase (Innovative Cell Technologies AT104-500) and cultured in Nutristem media with 10 µM Y-27632 (STEMCELL Technologies 72302). iPSCs were cultured to 70–90% confluency and cultured in RPMI-1640 supplemented with 1X B27 minus insulin (Thermo Fisher Scientific A1895601) and 4-6 µM CHIR-99021 (Selleck S2924) to initiate differentiation. Two days later, cells were refreshed with RPMI-1640 supplemented with 1X B27 minus insulin and 2 µM Wnt-C59 (Selleck S7037). Two days later, cells were refreshed in RPMI-1640 supplemented with 1× B27 minus insulin. Two days later, cells were refreshed in RPMI-1640 supplemented with 1× B27 (Thermo Fisher Scientific 17504-044) and maintained in this culture media for 4 days with a media change every other day. On day 10, cells were harvested with 80% Accutase and 20% TrypLE (Thermo Fisher Scientific A12177-01) and replated on Matrigel-coated plates in RPMI-1640 supplemented with 1× B27, 5% KnockOut Serum Replacement (Thermo Fisher Scientific 10828028), and 5 µM Y-27632. The following day, cells were refreshed with RPMI-1640 supplemented with 1× B27. From day 12 of differentiation onward, iPSC-CMs were maintained in RPMI-1640 minus glucose supplemented with 1× B27 and 4 mM lactate (Sigma-Aldrich L7900). Media was changed every two days. For the calcium flux and viability assays, cells were transduced with TNNT2-zeocin on day 15 of differentiation to select cardiomyocytes over fibroblasts. On day 25 of differentiation, iPSC-CMs were harvested with 80% Accutase and 20% TrypLE and replated on Matrigel-coated plates in RPMI-1640 supplemented with 1× B27, 5% KnockOut Serum Replacement, and 5 µM Y-27632. For monolayers, 1250 iPSC-CMs per 30 mm^2^ were seeded onto Matrigel-coated glass-bottomed 96-well plates (Cellvis P96-1.5H-N), and for the single-cell cultures, 20,000 iPSC-CMs per cm^2^ of the micropatterned area were seeded onto glass-bottomed 6-well plates (Cellvis P06-1.5H-N). Assays were performed between days 30 and 60 of differentiation.

### Immunofluorescence

For immunofluorescence staining, monolayer and single-cell iPSC-CMs were fixed on day 30 post differentiation in 4% paraformaldehyde in phosphate-buffered saline (PBS) for 15 min. iPSCs were fixed at the pluripotent state. Fixed samples were stored short-term in PBS at 4 °C. Fixed samples were blocked in 4% goat serum, 0.1% Triton X-100 in PBS for 1 h at room temperature. Cells were washed with PBS three times for 5 min and permeabilized in 0.1% Triton X-100 in PBS for 15 min. Cells were stained with the following antibodies: rabbit monoclonal ERα antibody (Thermo Fisher MA5-14501; 1:50 dilution), rabbit monoclonal beta-dystroglycan antibody (Abcam 43125; 1:200 dilution), mouse monoclonal cardiac troponin T antibody (Thermo Fisher MA5-12960, 1:200 dilution), mouse monoclonal dystrophin antibody (DSHB MANEX1A; 1:200 dilution) for the first pair of lines and rabbit polyclonal dystrophin antibody (Abcam 15277; 1:200 dilution) for the second pair of lines, mouse monoclonal OCT4 antibody (Millipore MAB4419; 1:100 dilution), mouse monoclonal SOX2 antibody conjugated to Alexa Fluor 488 (BD Pharmingen 561593; 1:100 dilution), or mouse monoclonal TRA-1-60 antibody (ESI-BIO ST11016; 1:100 dilution). Cells were incubated in primary antibodies overnight at 4 °C. After 3 ×5-min PBS washes, the following secondary antibodies were used: goat anti-rabbit Alexa 488 (Jackson ImmunoResearch 111-546-045), goat anti-mouse Alexa 488 (Jackson ImmunoResearch 115-546-072), goat anti-rabbit Alexa 647 (Jackson ImmunoResearch 111-606-045), and goat anti-mouse Alexa 647 (Jackson ImmunoResearch 115-605-207). Cells were incubated in secondary antibodies at a 1:500 dilution for 1 h at room temperature. After 3 × 5-min PBS washes, samples were mounted using ProLong Glass Antifade Mountant with NucBlue Stain (Thermo Fisher Scientific P36981) or were stained with 3 nM DAPI and mounted with Fluoromount-G (Southern Biotech 0100-01). In addition, on day 30 post differentiation, cells were either fixed first and then stained or stained directly with SiR-actin dye (Spirochrome CY-SC001) and Hoechst 33342 nuclear dye (Thermo Fisher Scientific H3570) according to the manufacturer’s protocols. Images were captured with 1920 × 1440 resolution using a ×10 or ×20 (for the monolayers) or a ×40 or ×63 (for the single cells) 0.17 numerical aperture CFI60 objective on a Keyence BZ-X710 microscope. Images were analyzed using CellProfiler. Confocal microscopy was performed on an Everest deconvolution workstation (Intelligent Imaging Innovations) equipped with a Zeiss AxioImager Z1 microscope and a CoolSnapHQ cooled CCD camera (Roper Scientific). Images were captured using a 63 × 1.4 numerical aperture Plan-Apochromat objective lens (Zeiss 420780-9900). Images were 3D deconvoluted with Microvolution.

### Reverse transcription-quantitative PCR

RNA was extracted with the Qiagen RNeasy Micro Kit, reverse transcription was performed with the Invitrogen SuperScript IV reverse transcriptase, and quantitative PCR was performed with the BioRad SsoAdvanced Univeral SYBR Green Supermix following the manufacturer’s instructions. The following primer sets were used: *MYBPC3* (forward: 5′-CAGCCGCTGAGCTGGGAG-3′; reverse: 5′-GCCACCCACGGTCACCTC-3′), *MYL2* (forward: 5′-TGCCCTTGGGCGAGTGAAC-3′; reverse: 5′-CAGCCTTCAGCACCCCTTTGC-3′), *MYH7* (forward: 5′-GACGGCACTGAAGAGGCTGAC-3′; reverse: 5′-CATACACTGCCTTGGCCAGTGC-3′), *TNNT2* (forward: 5′-GAGACCAGGGCAGAAGAAGATGAAGAAG-3′; reverse: 5′-ATCGGGGATCTTGGGAGGCAC-3′), *TNNT2* adult isoform (5′-GCAGCTGTTGAAGAGCAGGAGG-3′; reverse: 5′-ATCGGGGATCTTGGGAGGCAC-3′), *TPM1* (forward: 5′-CAGCAGATGAGAGTGAGAGAGGCATG-3′; reverse: 5′-GGCCACCTCTTCATATTTGCGGTC-3′), and *GAPDH* was performed (forward: 5′-GTGGACCTGACCTGCCGTC-3′; reverse: 5′-GGAGACCACCTGGTGCTCAG-3′).

### Microcontact printing

To induce the single iPSC-CMs to adopt a physiological, elongated shape, we used microcontact printing following published protocols^38^. Polydimethylsiloxane stamps were prepared by casting Sylgard 184 silicone elastomer mixture (Electron Microscopy Sciences 24236-10) onto silicon wafer molds with 7:1 length to width aspect ratio patterns and cured overnight at 65 °C. The stamps were cut out, washed with 70% ethanol, and air-dried. Stamps were coated with 100 μL of Matrigel diluted 1:10 and incubated overnight at 4 °C. Excess Matrigel was removed, and the coated stamps were blow-dried with compressed air. Stamps were compressed by 100 g weights onto glass-bottom wells for microcontact printing.

### Cardiomyocyte contractility assay

Spontaneous contractions of iPSC-CMs were recorded after treatment with isoproterenol (Sigma-Aldrich I5627-5G) or 4-hydroxytamoxifen (Cayman Chemicals 17308). For chronic treatment experiments, iPSC-CMs were refreshed every 3 days with media containing 0.5 µM 4-hydroxytamoxifen with ethanol as the vehicle. Stock solutions of drugs were diluted 1:1000 in media. Cells were imaged every 3 days for 30 days. For the monolayer experiments, 10-s videos were recorded in 3–6 regions of interest in each cell culture well, and for the single-cell experiments, 10-s videos of a minimum of 20 single cardiomyocytes were recorded in each cell culture well. Videos were captured at 29 fps with 760 × 920 resolution using a ×10 (monolayers) or a ×40 (single cells) 0.17 numerical aperture CFI60 objective (Nikon) in oblique brightfield video mode on a BZ-X710 microscope (Keyence). Videos were analyzed using live-cell video analysis software that measures beating rate and velocity^40^. In addition to contractility measurements, we assessed the duration of spontaneous contractions by calculating the percentage of beating cells over the time course of each single-cell experiment.

### Calcium flux assay

For ratiometric calcium imaging, we imaged Fura Red, AM (Thermo Fisher F3012) on the Zeiss AxioObserver inverted microscope and a Photometrics PRIME 95B sCMOS camera, using a custom-made filter cube with dual-band excitation filter (430/24 nm and 470/40 nm), dichronic mirror 505 nm and emission filter 670/50 nm (Chroma), and a Colibri 7 Type R fast switching LED illumination light source. Pseudo-simultaneous videos were recorded in a longitudinally-cropped area along the cardiomyocyte main axis using excitation wavelengths of 471 nm and 435 nm and emission wavelengths of 670 nm and 660 nm at 115 fps/channel for 14 s. Cells were paced at 1 Hz and maintained at 37 °C with 5% CO_2_ in a humidified chamber. Since Fura Red is a calcium quenching dye, ratiometric signals were calculated as signal at 471 nm/signal at 435 nm. Videos were analyzed using a custom MATLAB script.

### Viability assay

Calcein Blue, AM (Thermo Fisher C34853) was used for viability assays at day 30 and day 42 after 12 days of chronic 0.5 µm 4-hydroxytamoxifen treatment. Images of the entire well were captured at ×10 objective on a Keyence microscope. Images capturing the entire well were stitched and analyzed in ImageJ.

### Statistical analysis

Statistical significance was determined using a two-tailed unpaired two-sample *t* test or one-way ANOVA followed by Tukey test for post hoc comparison for more than two conditions. Distributions were assessed for normality and lognormality prior to performing the statistical tests. Log-rank (Mantel–Cox) test with Bonferroni correction was used to determine pairwise differences in Kaplan–Meier curves. Pearson’s chi-square test was used to determine significant differences in frequencies between different groups.

### Reporting summary

Further information on research design is available in the Nature Research Reporting Summary linked to this article.

## Supplementary information

Supplementary Information

REPORTING SUMMARY

## Data Availability

The datasets generated in the current study are available upon request from the corresponding authors.

## Code availability

Custom MATLAB code used for calcium analysis is available upon request from the corresponding authors.
