# Supplementary material for: Tamoxifen treatment ameliorates contractile dysfunction of Duchenne muscular dystrophy stem cell-derived cardiomyocytes on bioengineered substrates
Source: NPJ Regen Med. 2022 Mar 18;7:19. doi: 10.1038/s41536-022-00214-x (PMC8933505; doi:10.1038/s41536-022-00214-x)
Supplement: Supplementary file 1 — Supplementary Information [file 41536_2022_214_MOESM1_ESM.pdf]

Tamoxifen treatment ameliorates contractile dysfunction of Duchenne muscular dystrophy stem cell-derived cardiomyocytes on bioengineered substrates

Foster Birnbaum, Asuka Eguchi, Gaspard Pardon, Alex C. Y. Chang, and Helen M. Blau

Supplementary Information

Supplementary Figure 1. Characterization of iPSCs.

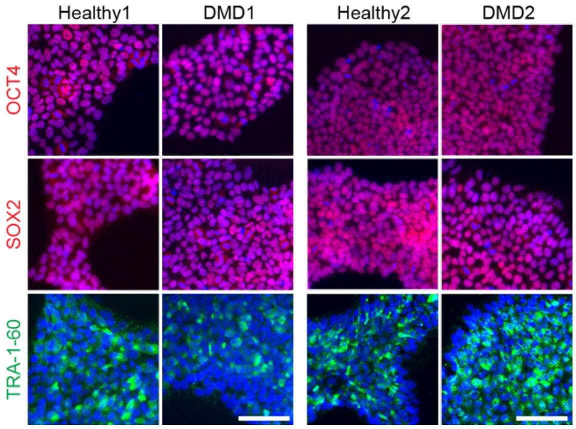

The pluripotency markers OCT4 (red), SOX2 (red), and TRA-1-60 (green) were immunostained in iPSCs. DAPI in blue marks the nuclei. Scale bar represents 100 μm.

Supplementary Figure 2. Markers of cardiomyocyte differentiation in iPSC-CMs.

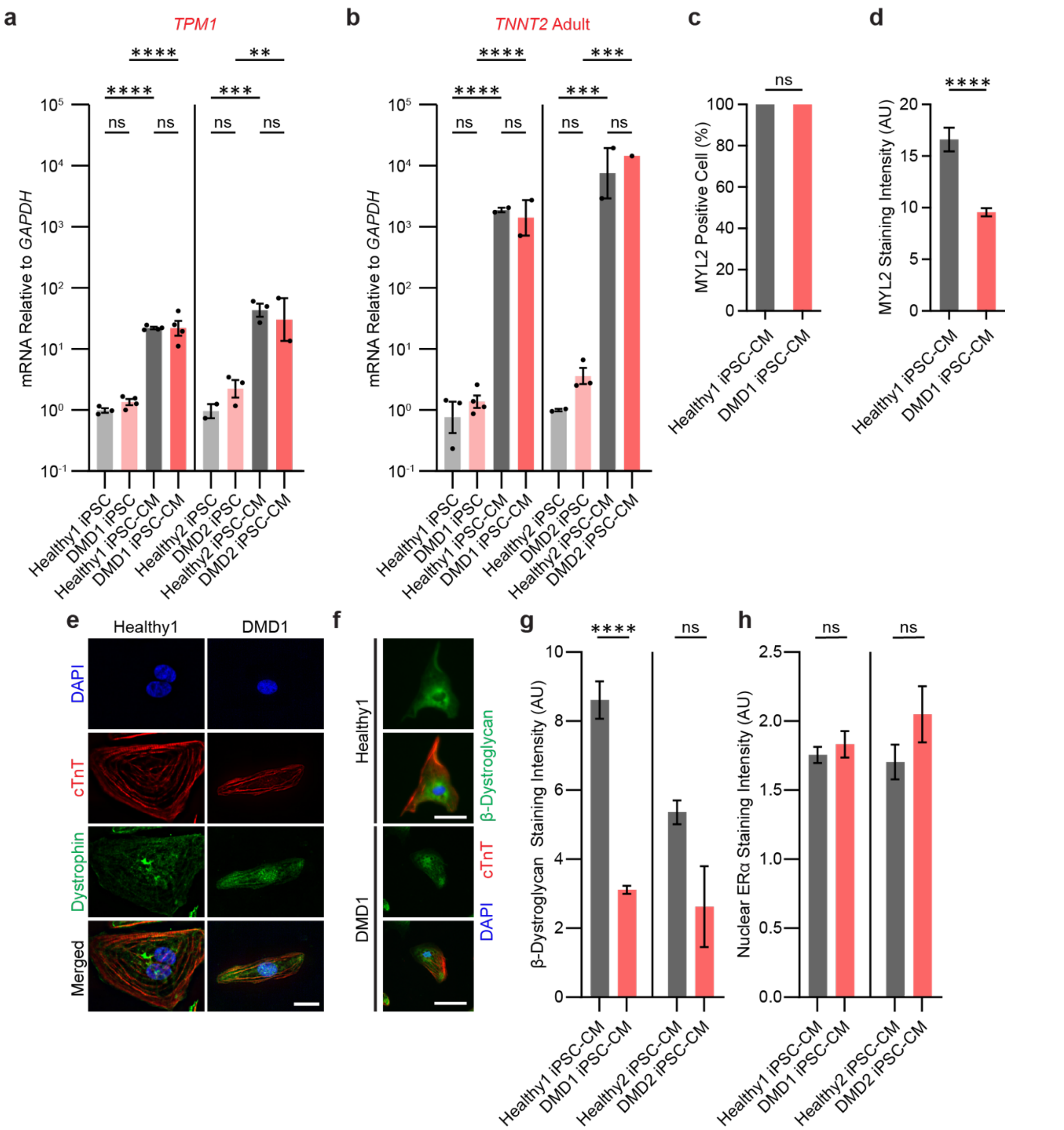

(**a, b**) RT-qPCR results in iPSCs and iPSC-CMs for *TPM1* and the adult isoform of *TNNT2* (i.e., without exon 5). Data represent mean ± SEM. Statistical significance determined by one-way ANOVA and Tukey test for post-hoc comparison. **$p < 0.01$, ***$p < 0.001$, ****$p < 0.0001$, ns (non-significant). n = 1-4 biological replicates. (**c**) Percentage of MYL2 positive cells. (**d**) MYL2 signal relative to cell area in arbitrary units. Data represent mean ± SEM. Statistical significance determined by unpaired t-test. ****$p < 0.0001$, ns (non-significant). n = 3 biological replicates. N = 34-131 cells. (**e**) 3D deconvoluted micrographs of iPSC-CMs stained for DAPI (blue), cTnT (red), and dystrophin (green) taken at 63X magnification. Healthy1 and DMD1 iPSC-CMs were stained with ab15277. (**f**) Micrographs of iPSC-CMs of beta-dystroglycan (green), cTnT (red), and DAPI (blue) staining taken at 40X magnification. Scale bars represent 20 µm. (**g**) Beta-dystroglycan signal relative to cell area in arbitrary units. (**h**) Nuclear signal relative to cytoplasmic signal of ERα staining in arbitrary units. Data represent mean ± SEM. Statistical significance determined by unpaired t-test. ***$p < 0.001$, ns (non-significant). n = 2-5 biological replicates. N = 172-19,127 cells.

Supplementary Figure 3. Raw data of beating rate and beating velocity in iPSC-CMs cultured in monolayers.

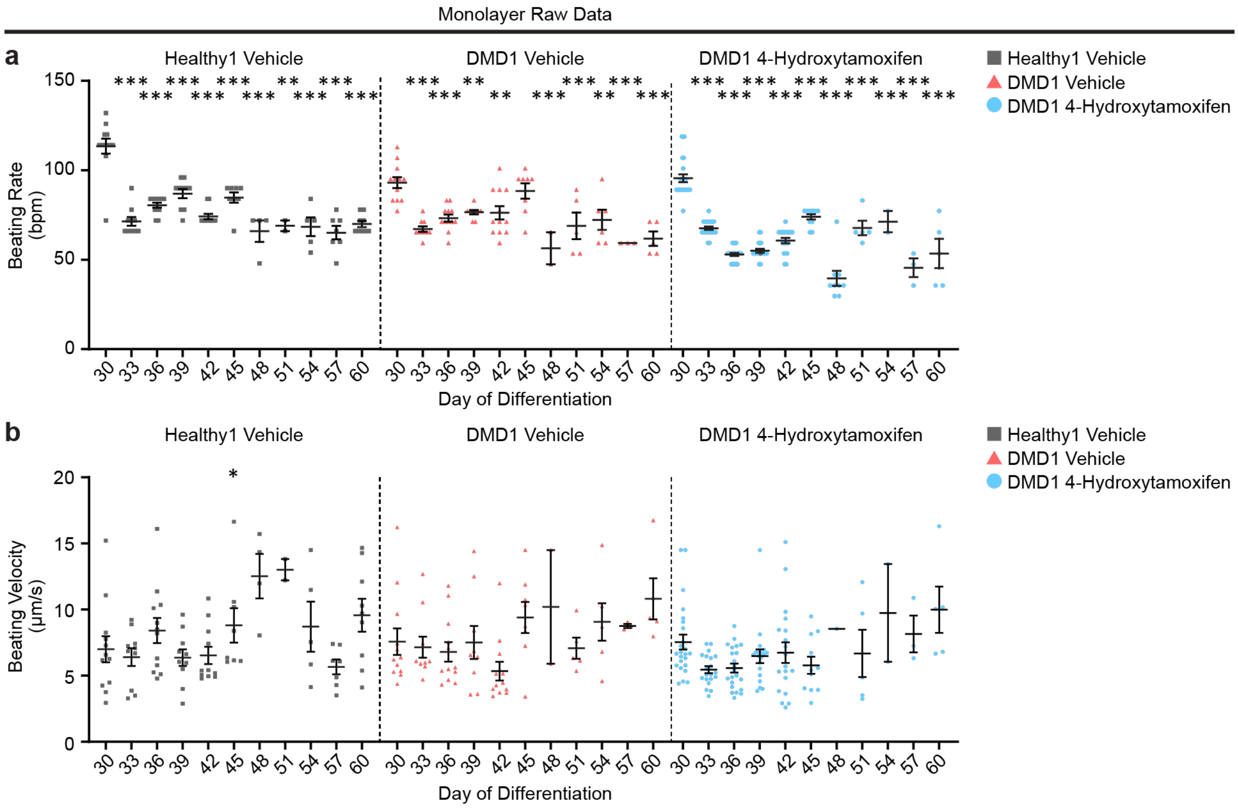

(**a**) Beating rate in beats per minute of iPSC-CMs. (**b**) Beating velocity in µm/s of iPSC-CMs. Cells were treated with vehicle or 0.5 µM 4-hydroxytamoxifen every 3 days beginning on Day 30 of differentiation. The same monolayer region of interest was tracked over time. Data represent mean ± SEM. Statistical significance compares day of treatment to pre-treatment (Day 0). Statistical significance determined by one-way ANOVA and Tukey test for post-hoc comparison. *$p < 0.05$, **$p < 0.01$, ***$p < 0.001$. n = 3 biological replicates, N = 2-24 cells.

Supplementary Figure 4. Beating rate and beating velocity for iPSC-CMs cultured in monolayers.

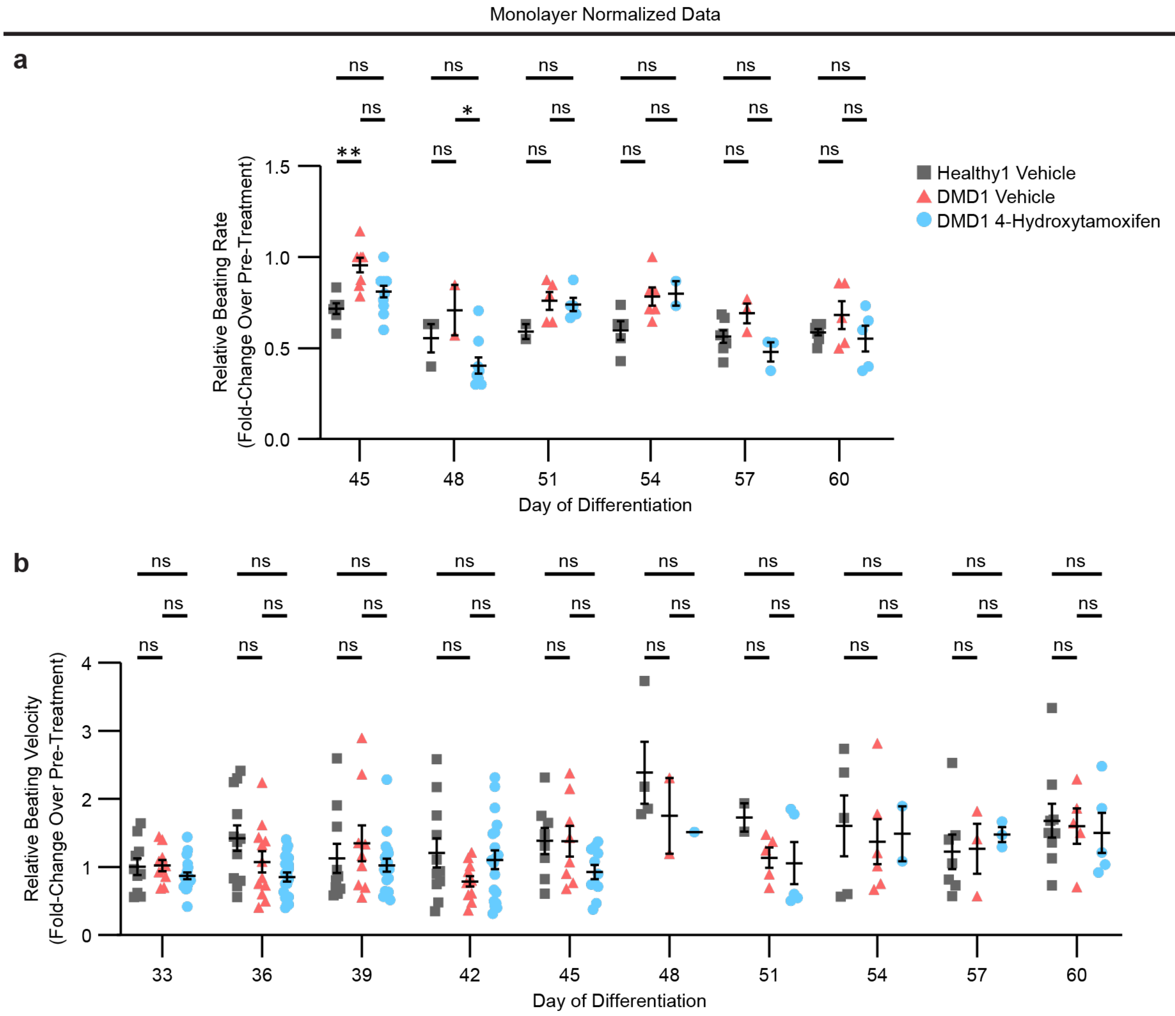

(**a**) Relative beating rate represented as fold-change over pre-treatment (Day 30) of iPSC-CMs cultured in monolayers from Day 45 to Day 60 of differentiation. n = 3 biological replicates, N = 2-11 cells. (**b**) Relative beating velocity represented as fold-change over pre-treatment (Day 30). Cells were treated with vehicle or 0.5 µM 4-hydroxytamoxifen every 3 days beginning on Day 30 of differentiation. The same monolayer region of interest was tracked over time. Data represent mean ± SEM. Statistical significance determined by one-way ANOVA and Tukey test for post-hoc comparison. $^{**}p < 0.01$, $^{***}p < 0.001$, ns (non-significant). n = 3 biological replicates, N = 1-22 cells.

Supplementary Figure 5. Raw data of beating rate and beating velocity in micropatterened iPSC-CMs as single cells.

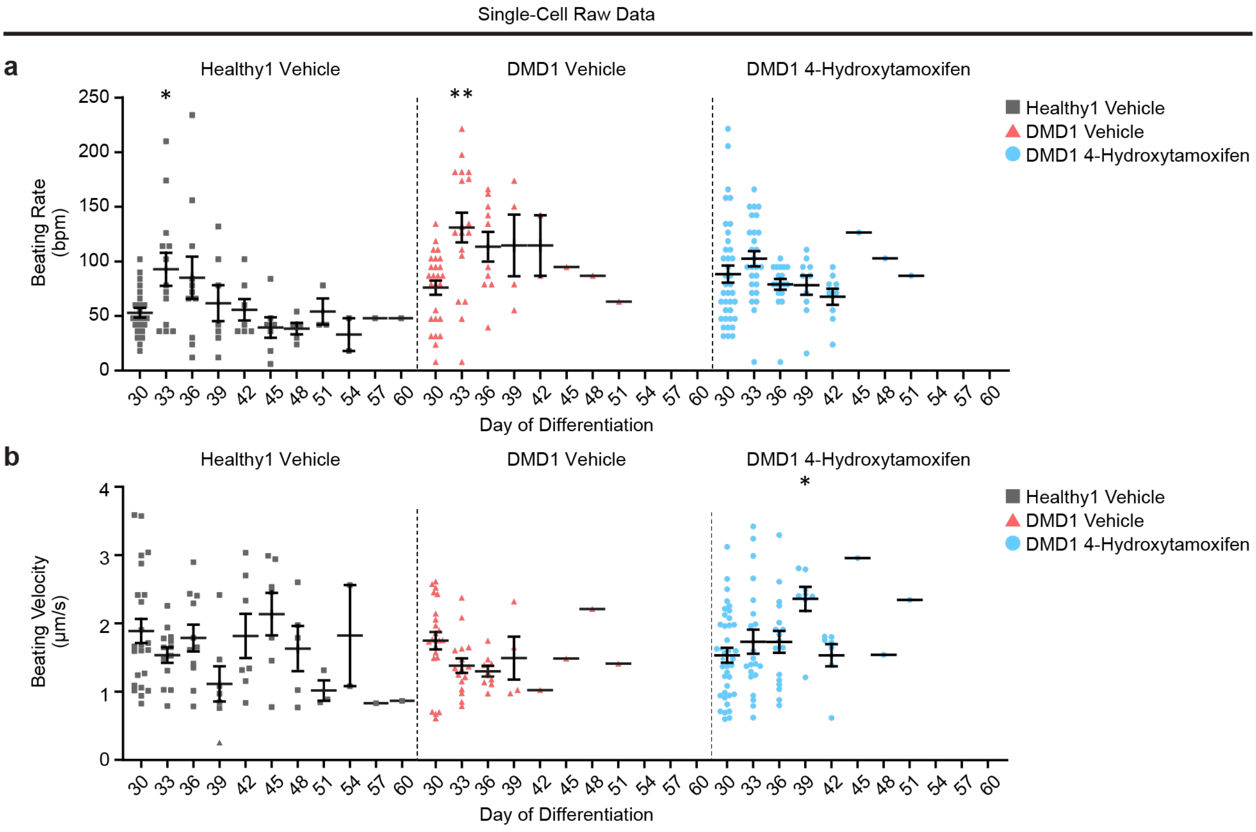

(**a**) Beating rate in beats per minute of iPSC-CMs. n = 3 biological replicates, N = 1-38 cells. (**b**) Beating velocity in μm/s of iPSC-CMs. Cells were treated with vehicle or 0.5 μM 4-hydroxytamoxifen every 3 days beginning on Day 30 of differentiation. Individual cells were tracked over time. Data represent mean ± SEM. Statistical significance compares Day of differentiation to pre-treatment (Day 30). n = 3 biological replicates, N = 1-36 cells. Statistical significance determined by one-way ANOVA and Tukey test for post-hoc comparison. *$p < 0.05$, **$p < 0.01$, ***$p < 0.001$.

Supplementary Figure 6. Calcium handling in micropatterned iPSC-CMs.

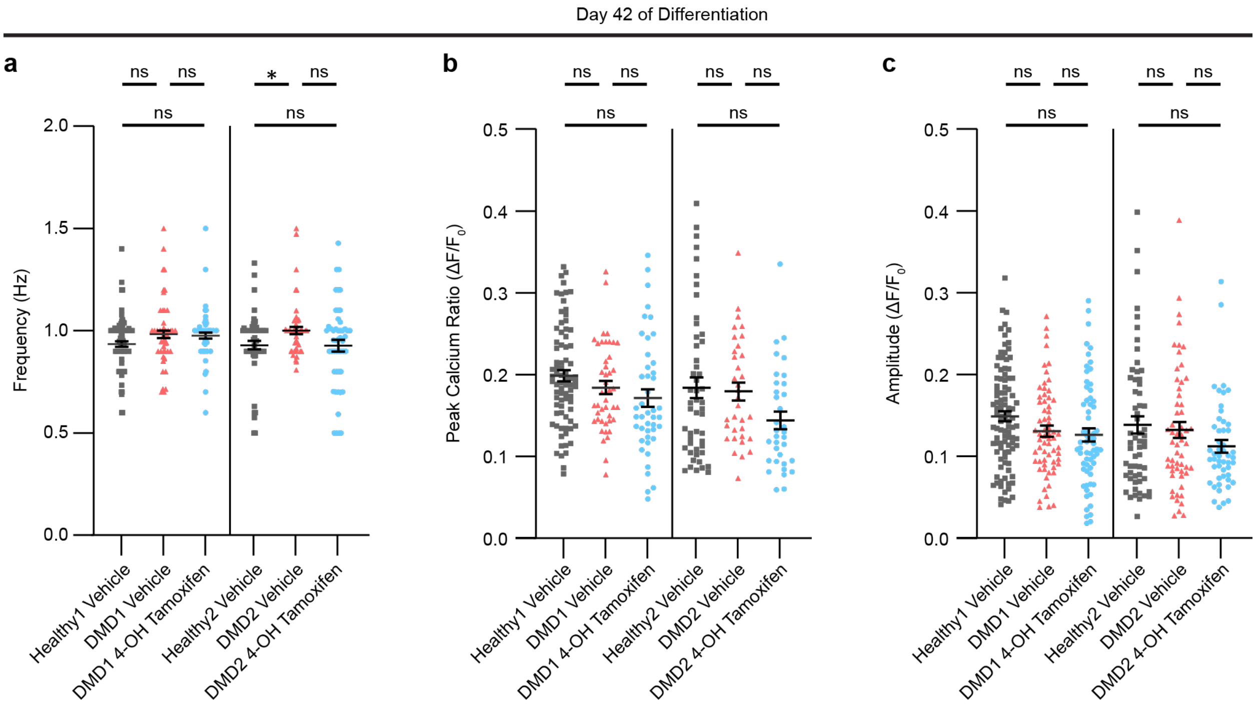

(**a, b, c**) Peak frequency, peak calcium ratio, and transient amplitude in iPSC-CMs on Day 42 of differentiation. Cells were treated with vehicle or 0.5 μM 4-hydroxytamoxifen every 3 days beginning on Day 30 of differentiation. Data represent mean ± SEM. Statistical significance determined by one-way ANOVA and Tukey test for post-hoc comparison. **p* < 0.05, ****p* < 0.001, ns (non-significant). n = 3 biological replicates, N = 34-99 cells.

Supplementary Table 1. Null hypothesis test results

Panel	Comparison	t	Mean Difference	SE of Mean Diff	n1	n2	df	P value	Test
Fig. 2a Line 1	log(Healthy1 iPSC) vs. log(Healthy1 iPSC-CM)	13.04	-3.369	0.2583	3	4	11	<0.0001	One-way ANOVA
	log(DMD1 iPSC) vs. log(DMD1 iPSC-CM)	14.42	-3.449	0.2392	4	4	11	<0.0001	One-way ANOVA
	log(Healthy1 iPSC-CM) vs. log(DMD1 iPSC-CM)	0.4833	-0.1156	0.2392	4	4	11	0.6383	One-way ANOVA
	log(Healthy1 iPSC) vs. log(DMD1 iPSC)	0.1363	-0.03522	0.2583	3	4	11	0.894	One-way ANOVA
Fig. 2a Line 2	log(Healthy2 iPSC) vs. log(DMD2 iPSC)	0.4343	0.1762	0.4057	2	3	6	0.6792	One-way ANOVA
	log(Healthy2 iPSC) vs. log(Healthy2 iPSC-CM)	7.793	-3.162	0.4057	2	3	6	0.0002	One-way ANOVA
	log(Healthy2 iPSC-CM) vs. log(DMD2 iPSC-CM)	1.378	-0.5589	0.4057	3	2	6	0.2175	One-way ANOVA
	log(DMD2 iPSC) vs. log(DMD2 iPSC-CM)	9.605	-3.897	0.4057	3	2	6	<0.0001	One-way ANOVA
Fig. 2b Line 1	log(Healthy1 iPSC) vs. log(Healthy1 iPSC-CM)	22.37	-4.342	0.1941	3	4	11	<0.0001	One-way ANOVA
	log(DMD1 iPSC) vs. log(DMD1 iPSC-CM)	20.97	-3.768	0.1797	4	4	11	<0.0001	One-way ANOVA
	log(Healthy1 iPSC-CM) vs. log(DMD1 iPSC-CM)	1.14	0.2049	0.1797	4	4	11	0.2784	One-way ANOVA
	log(Healthy1 iPSC) vs. log(DMD1 iPSC)	1.9	-0.3688	0.1941	3	4	11	0.0839	One-way ANOVA
Fig. 2b Line 2	log(Healthy2 iPSC) vs. log(DMD2 iPSC)	1.896	-0.9103	0.4801	2	3	6	0.1068	One-way ANOVA
	log(Healthy2 iPSC) vs. log(Healthy2 iPSC-CM)	10.88	-5.224	0.4801	2	3	6	<0.0001	One-way ANOVA
	log(Healthy2 iPSC-CM) vs. log(DMD2 iPSC-CM)	2.287	1.098	0.4801	3	2	6	0.0622	One-way ANOVA
	log(DMD2 iPSC) vs. log(DMD2 iPSC-CM)	6.697	-3.215	0.4801	3	2	6	0.0005	One-way ANOVA
Fig. 2c Line 1	log(Healthy1 iPSC) vs. log(Healthy1 iPSC-CM)	17.47	-5.202	0.2977	3	4	11	<0.0001	One-way ANOVA
	log(DMD1 iPSC) vs. log(DMD1 iPSC-CM)	15.3	-4.216	0.2756	4	4	11	<0.0001	One-way ANOVA
	log(Healthy1 iPSC-CM) vs. log(DMD1 iPSC-CM)	0.2605	0.07182	0.2756	4	4	11	0.9984	One-way ANOVA
	log(Healthy1 iPSC) vs. log(DMD1 iPSC)	3.069	-0.9138	0.2977	3	4	11	0.042	One-way ANOVA
Fig. 2c Line 2	log(Healthy2 iPSC) vs. log(DMD2 iPSC)	0.8492	-0.3079	0.3626	2	3	6	0.8932	One-way ANOVA
	log(Healthy2 iPSC) vs. log(Healthy2 iPSC-CM)	14.22	-5.158	0.3626	2	3	6	<0.0001	One-way ANOVA
	log(Healthy2 iPSC-CM) vs. log(DMD2 iPSC-CM)	0.1819	0.06597	0.3626	3	2	6	0.9996	One-way ANOVA
	log(DMD2 iPSC) vs. log(DMD2 iPSC-CM)	13.19	-4.784	0.3626	3	2	6	<0.0001	One-way ANOVA

Panel	Comparison	t	Mean Difference	SE of Mean Diff	n1	n2	df	P value	Test
Fig. 2d Line 1	log(Healthy1 iPSC) vs. log(Healthy1 iPSC-CM)	18.14	-3.056	0.1684	3	4	11	<0.0001	One-way ANOVA
	log(DMD1 iPSC) vs. log(DMD1 iPSC-CM)	18.15	-2.83	0.1559	4	4	11	<0.0001	One-way ANOVA
	log(Healthy1 iPSC-CM) vs. log(DMD1 iPSC-CM)	0.2023	-0.03155	0.1559	4	4	11	0.8434	One-way ANOVA
	log(Healthy1 iPSC) vs. log(DMD1 iPSC)	1.53	-0.2577	0.1684	3	4	11	0.1543	One-way ANOVA
Fig. 2d Line 2	log(Healthy2 iPSC) vs. log(DMD2 iPSC)	1.085	-0.5822	0.5368	2	3	6	0.3197	One-way ANOVA
	log(Healthy2 iPSC) vs. log(Healthy2 iPSC-CM)	6.69	-3.591	0.5368	2	3	6	0.0005	One-way ANOVA
	log(Healthy2 iPSC-CM) vs. log(DMD2 iPSC-CM)	2.072	-1.112	0.5368	3	2	6	0.0837	One-way ANOVA
	log(DMD2 iPSC) vs. log(DMD2 iPSC-CM)	7.677	-4.121	0.5368	3	2	6	0.0003	One-way ANOVA
Fig. 2h	Healthy1 vs. DMD1	2.351	-0.5657	0.2406	9996	9235	19229	0.0187	Two-tailed unpaired t test
	Healthy2 vs. DMD2	0.4043	0.1837	0.4545	4227	2818	7043	0.6860	Two-tailed unpaired t test
Fig. 3e	Vehicle vs. 1 nM isoproterenol	2.453	-0.3019	0.1231	17	15	56	0.0614	One-way ANOVA
	Vehicle vs. 5 nM isoproterenol	4.962	-0.65	0.131	17	12	56	<0.0001	One-way ANOVA
	Vehicle vs. 10 nM isoproterenol	4.879	-0.6559	0.1344	17	11	56	<0.0001	One-way ANOVA
	Vehicle vs. 50 nM isoproterenol	3.06	-0.5048	0.165	17	6	56	0.0128	One-way ANOVA
Fig. 3f	Vehicle vs. 1 nM isoproterenol	1.56	-0.2193	0.1406	13	24	55	0.2835	One-way ANOVA
	Vehicle vs. 10 nM isoproterenol	2.01	-0.3161	0.1573	13	14	55	0.1219	One-way ANOVA
	Vehicle vs. 50 nM isoproterenol	2.46	-0.4513	0.1835	13	8	55	0.0448	One-way ANOVA
Fig. 4a	(Day 3) Healthy1 Vehicle vs DMD1 Vehicle	2.786	-0.1308	0.04694	10	10	230	0.1595	One-way ANOVA
	(Day 3) Healthy1 Vehicle vs DMD1 4-OH Tam	2.525	-0.1018	0.04032	10	21	230	0.309	One-way ANOVA
	(Day 3) DMD1 Vehicle vs DMD1 4-OH Tam	0.7184	0.02897	0.04032	10	21	230	>0.9999	One-way ANOVA
	(Day 6) Healthy1 Vehicle vs DMD1 Vehicle	2.586	-0.1133	0.04381	11	12	230	0.2675	One-way ANOVA
	(Day 6) Healthy1 Vehicle vs DMD1 4-OH Tam	3.224	0.1249	0.03876	11	22	230	0.0426	One-way ANOVA
	(Day 6) DMD1 Vehicle vs DMD1 4-OH Tam	6.325	0.2382	0.03767	12	22	230	<0.0001	One-way ANOVA
	(Day 9) Healthy1 Vehicle vs DMD1 Vehicle	1.995	-0.09871	0.04948	9	9	230	0.7657	One-way ANOVA
	(Day 9) Healthy1 Vehicle vs DMD1 4-OH Tam	3.658	0.1553	0.04247	9	19	230	0.0094	One-way ANOVA

Panel	Comparison	t	Mean Difference	SE of Mean Diff	n1	n2	df	P value	Test
Fig. 4a cont.	(Day 9) DMD1 Vehicle vs DMD1 4-OH Tam	5.982	0.254	0.04247	9	19	230	<0.0001	One-way ANOVA
	(Day 12) Healthy1 Vehicle vs DMD1 Vehicle	4.199	-0.1887	0.04494	10	12	230	0.0011	One-way ANOVA
	(Day 12) Healthy1 Vehicle vs DMD1 4-OH Tam	0.2572	-0.01055	0.041	10	19	230	>0.9999	One-way ANOVA
	(Day 12) DMD1 Vehicle vs DMD1 4-OH Tam	4.604	0.1782	0.0387	12	19	230	0.0002	One-way ANOVA
Fig. 4b Line 1	Healthy1 Vehicle vs DMD1 Vehicle	8.965	38.14	4.254	3	5	9	<0.0001	One-way ANOVA
	Healthy1 Vehicle vs DMD1 4-OH Tam	5.834	25.96	4.449	3	4	9	0.0007	One-way ANOVA
	DMD1 Vehicle vs DMD1 4-OH Tam	3.118	-12.18	3.907	5	4	9	0.0366	One-way ANOVA
Fig. 4b Line 2	Healthy2 Vehicle vs DMD2 Vehicle	10.45	64.52	6.176	4	5	11	<0.0001	One-way ANOVA
	Healthy2 Vehicle vs DMD2 4-OH Tam	7.557	46.67	6.176	4	5	11	<0.0001	One-way ANOVA
	DMD2 Vehicle vs DMD2 4-OH Tam	3.066	-17.85	5.823	5	5	11	0.0319	One-way ANOVA
Fig. 5c	Healthy1 Vehicle vs DMD1 Vehicle	1.686	-0.5347	0.3172	10	10	35	0.1008	One-way ANOVA
	Healthy1 Vehicle vs DMD1 4-OH Tam	0.3446	0.09641	0.2798	10	18	35	0.7324	One-way ANOVA
	DMD1 Vehicle vs DMD1 4-OH Tam	2.256	0.6311	0.2798	10	18	35	0.0304	One-way ANOVA
Fig. 5d	Healthy1 Vehicle vs DMD1 Vehicle	1.507	0.4658	0.309	11	9	35	0.1407	One-way ANOVA
	Healthy1 Vehicle vs DMD1 4-OH Tam	0.7197	-0.1894	0.2631	11	18	35	0.4765	One-way ANOVA
	DMD1 Vehicle vs DMD1 4-OH Tam	2.334	-0.6551	0.2806	9	18	35	0.0254	One-way ANOVA
Fig. 6e Line 1	Healthy1 Vehicle vs. DMD1 Vehicle	2.182	-0.02212	0.01014	99	65	225	0.0877	One-way ANOVA
	Healthy1 Vehicle vs. DMD1 4-OH Tam	2.071	-0.02109	0.01018	99	64	225	0.114	One-way ANOVA
	DMD1 Vehicle vs DMD1 4-OH Tam	0.09241	0.001033	0.01118	65	64	225	0.9996	One-way ANOVA
Fig. 6e Line 2	Healthy2 Vehicle vs. DMD2 Vehicle	2.37	-0.03654	0.01542	59	57	163	0.0558	One-way ANOVA
	Healthy2 Vehicle vs. DMD2 4-OH Tam	0.3827	0.006107	0.01596	59	50	163	0.9736	One-way ANOVA
	DMD2 Vehicle vs DMD2 4-OH Tam	2.652	0.04265	0.01608	57	50	163	0.0262	One-way ANOVA
Fig. 6f Line 1	Healthy1 Vehicle vs. DMD1 Vehicle	2.208	-0.00426	0.00192	75	56	180	0.0832	One-way ANOVA
	Healthy1 Vehicle vs. DMD1 4-OH Tam	1.747	0.003443	0.00197	75	52	180	0.2273	One-way ANOVA
	DMD1 Vehicle vs DMD1 4-OH Tam	3.661	0.007702	0.00210	56	52	180	0.001	One-way ANOVA

Panel	Comparison	t	Mean Difference	SE of Mean Diff	n1	n2	df	P value	Test
Fig. 6f Line 2	Healthy2 Vehicle vs. DMD2 Vehicle	1.465	-0.00405	0.00276	50	35	114	0.3765	One-way ANOVA
	Healthy2 Vehicle vs. DMD2 4-OH Tam	1.084	0.003327	0.00306	35	32	114	0.6277	One-way ANOVA
	DMD2 Vehicle vs DMD2 4-OH Tam	0.2549	-0.00072	0.00284	50	32	114	0.9919	One-way ANOVA
Fig. S2a Line 1	log(Healthy1 iPSC) vs. log(Healthy1 iPSC-CM)	12.56	-1.348	0.1074	3	4	11	<0.0001	One-way ANOVA
	log(DMD1 iPSC) vs. log(DMD1 iPSC-CM)	12.16	-1.208	0.09942	4	4	11	<0.0001	One-way ANOVA
	log(Healthy1 iPSC-CM) vs. log(DMD1 iPSC-CM)	0.04416	0.00439	0.09942	4	4	11	0.9656	One-way ANOVA
	log(Healthy1 iPSC) vs. log(DMD1 iPSC)	1.261	-0.1355	0.1074	3	4	11	0.2332	One-way ANOVA
Fig. S2a Line 2	log(Healthy2 iPSC) vs. log(DMD2 iPSC)	1.45	-0.3678	0.2537	2	3	6	0.1973	One-way ANOVA
	log(Healthy2 iPSC) vs. log(Healthy2 iPSC-CM)	6.51	-1.652	0.2537	2	3	6	0.0006	One-way ANOVA
	log(Healthy2 iPSC-CM) vs. log(DMD2 iPSC-CM)	0.6121	0.1553	0.2537	3	2	6	0.563	One-way ANOVA
	log(DMD2 iPSC) vs. log(DMD2 iPSC-CM)	4.449	-1.128	0.2537	3	2	6	0.0043	One-way ANOVA
Fig. S2b Line 1	log(Healthy1 iPSC) vs. log(Healthy1 iPSC-CM)	11.83	-3.392	0.2867	3	2	7	<0.0001	One-way ANOVA
	log(DMD1 iPSC) vs. log(DMD1 iPSC-CM)	11.06	-3.01	0.272	4	2	7	<0.0001	One-way ANOVA
	log(Healthy1 iPSC-CM) vs. log(DMD1 iPSC-CM)	0.4055	0.1274	0.3141	2	2	7	0.6972	One-way ANOVA
	log(Healthy1 iPSC) vs. log(DMD1 iPSC)	1.064	-0.2552	0.2399	3	4	7	0.3228	One-way ANOVA
Fig. S2b Line 2	log(Healthy2 iPSC) vs. log(DMD2 iPSC)	1.833	-0.5579	0.3044	2	3	4	0.1407	One-way ANOVA
	log(Healthy2 iPSC) vs. log(Healthy2 iPSC-CM)	11.64	-3.88	0.3334	2	2	4	0.0003	One-way ANOVA
	log(Healthy2 iPSC-CM) vs. log(DMD2 iPSC-CM)	0.6911	-0.2822	0.4083	2	1	4	0.5275	One-way ANOVA
	log(DMD2 iPSC) vs. log(DMD2 iPSC-CM)	9.362	-3.604	0.385	3	1	4	0.0007	One-way ANOVA
Fig. S2c	Healthy1 iPSC-CM vs. DMD1 iPSC-CM	0.000	0.000	0.9757	34	131	163	>0.9999	Two-tailed unpaired t test
Fig. S2d	Healthy1 iPSC-CM vs. DMD1 iPSC-CM	7.230	-7.054	0.9757	34	131	163	<0.0001	Two-tailed unpaired t test

Panel	Comparison	t	Mean Difference	SE of Mean Diff	n1	n2	df	P value	Test
Fig. S2g	Healthy1 vs. DMD1	4.436	-5.497	1.239	172	19127	19297	<0.0001	Two-tailed unpaired t test
	Healthy2 vs. DMD2	0.6267	-2.738	4.369	740	10265	11003	0.5309	Two-tailed unpaired t test
Fig. S2h	Healthy1 vs. DMD1	0.6970	0.07670	0.1100	9996	9235	19229	0.4858	Two-tailed unpaired t test
	Healthy2 vs. DMD2	1.286	0.3459	0.2690	2818	4227	7043	0.1985	Two-tailed unpaired t test
Fig. S3a Healthy1 Vehicle	Day 0 vs Day 3	11.07	42.1	3.804	12	10	79	<0.0001	One-way ANOVA
	Day 0 vs Day 6	9.098	33	3.627	12	12	79	<0.0001	One-way ANOVA
	Day 0 vs Day 9	6.966	26.5	3.804	12	10	79	<0.0001	One-way ANOVA
	Day 0 vs Day 12	10.6	39.32	3.709	12	11	79	<0.0001	One-way ANOVA
	Day 0 vs Day 15	7.09	28.75	4.055	12	8	79	<0.0001	One-way ANOVA
	Day 0 vs Day 18	9.26	47.5	5.129	12	4	79	<0.0001	One-way ANOVA
	Day 0 vs Day 21	6.558	44.5	6.786	12	2	79	<0.0001	One-way ANOVA
	Day 0 vs Day 24	9.537	45.1	4.729	12	5	79	<0.0001	One-way ANOVA
	Day 0 vs Day 27	11.44	48.36	4.225	12	7	79	<0.0001	One-way ANOVA
	Day 0 vs Day 30	11.1	43.5	3.918	12	9	79	<0.0001	One-way ANOVA
Fig. S3a DMD1 Vehicle	Day 0 vs Day 3	6.004	26.2	4.364	12	10	73	<0.0001	One-way ANOVA
	Day 0 vs Day 6	4.807	20	4.16	12	12	73	<0.0001	One-way ANOVA
	Day 0 vs Day 9	3.709	16.67	4.494	12	9	73	0.004	One-way ANOVA
	Day 0 vs Day 12	4.086	17	4.16	12	12	73	0.0011	One-way ANOVA
	Day 0 vs Day 15	1.021	4.75	4.652	12	8	73	0.9757	One-way ANOVA
	Day 0 vs Day 18	4.754	37	7.783	12	2	73	<0.0001	One-way ANOVA
	Day 0 vs Day 21	4.498	24.4	5.425	12	5	73	0.0003	One-way ANOVA
	Day 0 vs Day 24	4.121	21	5.095	12	6	73	0.001	One-way ANOVA

Panel	Comparison	t	Mean Difference	SE of Mean Diff	n1	n2	df	P value	Test
Fig. S3a DMD1 Vehicle cont.	Day 0 vs Day 27	5.169	34	6.578	12	3	73	<0.0001	One-way ANOVA
	Day 0 vs Day 30	5.825	31.6	5.425	12	5	73	<0.0001	One-way ANOVA
Fig. S3a DMD1 4-OH Tam	Day 0 vs Day 3	11.78	28.21	2.394	24	21	129	<0.0001	One-way ANOVA
	Day 0 vs Day 6	18.2	43.05	2.365	24	22	129	<0.0001	One-way ANOVA
	Day 0 vs Day 9	16.63	40.92	2.46	24	19	129	<0.0001	One-way ANOVA
	Day 0 vs Day 12	14.32	35.24	2.46	24	19	129	<0.0001	One-way ANOVA
	Day 0 vs Day 15	7.463	21.77	2.917	24	11	129	<0.0001	One-way ANOVA
	Day 0 vs Day 18	18.04	56.5	3.132	24	9	129	<0.0001	One-way ANOVA
	Day 0 vs Day 21	7.134	28.1	3.939	24	5	129	<0.0001	One-way ANOVA
	Day 0 vs Day 24	4.155	24.5	5.897	24	2	129	0.0006	One-way ANOVA
	Day 0 vs Day 27	10.29	50.5	4.907	24	3	129	<0.0001	One-way ANOVA
	Day 0 vs Day 30	10.79	42.5	3.939	24	5	129	<0.0001	One-way ANOVA
Fig. S3b Healthy1 Vehicle	Day 0 vs Day 3	0.4721	0.6016	1.274	12	10	79	>0.9999	One-way ANOVA
	Day 0 vs Day 6	1.157	-1.405	1.215	12	12	79	0.9443	One-way ANOVA
	Day 0 vs Day 9	0.5035	0.6416	1.274	12	10	79	>0.9999	One-way ANOVA
	Day 0 vs Day 12	0.3797	0.4716	1.242	12	11	79	>0.9999	One-way ANOVA
	Day 0 vs Day 15	1.327	-1.803	1.358	12	8	79	0.8757	One-way ANOVA
	Day 0 vs Day 18	3.211	-5.517	1.718	12	4	79	0.019	One-way ANOVA
	Day 0 vs Day 21	2.644	-6.011	2.273	12	2	79	0.0944	One-way ANOVA
	Day 0 vs Day 24	1.072	-1.698	1.584	12	5	79	0.966	One-way ANOVA
	Day 0 vs Day 27	0.9517	1.347	1.415	12	7	79	0.9853	One-way ANOVA
	Day 0 vs Day 30	1.954	-2.564	1.312	12	9	79	0.4276	One-way ANOVA
Fig. S3b DMD1 Vehicle	Day 0 vs Day 3	0.3224	0.4195	1.301	12	10	73	>0.9999	One-way ANOVA
	Day 0 vs Day 6	0.6242	0.7744	1.241	12	12	73	0.9995	One-way ANOVA
	Day 0 vs Day 9	0.04434	0.05941	1.34	12	9	73	>0.9999	One-way ANOVA
	Day 0 vs Day 12	1.797	2.229	1.241	12	12	73	0.5486	One-way ANOVA

Panel	Comparison	t	Mean Difference	SE of Mean Diff	n1	n2	df	P value	Test
Fig. S3b DMD1 Vehicle cont.	Day 0 vs Day 15	1.32	-1.831	1.387	12	8	73	0.8796	One-way ANOVA
	Day 0 vs Day 18	1.133	-2.629	2.321	12	2	73	0.9515	One-way ANOVA
	Day 0 vs Day 21	0.3075	0.4973	1.618	12	5	73	>0.9999	One-way ANOVA
	Day 0 vs Day 24	0.9849	-1.496	1.519	12	6	73	0.9812	One-way ANOVA
	Day 0 vs Day 27	0.6046	-1.186	1.962	12	3	73	0.9996	One-way ANOVA
	Day 0 vs Day 30	2.006	-3.244	1.618	12	5	73	0.3924	One-way ANOVA
Fig. S3b DMD1 4-OH Tam	Day 0 vs Day 3	2.726	2.086	0.7652	24	20	120	0.0714	One-way ANOVA
	Day 0 vs Day 6	2.632	1.963	0.746	24	22	120	0.092	One-way ANOVA
	Day 0 vs Day 9	1.371	1.064	0.7761	24	19	120	0.8504	One-way ANOVA
	Day 0 vs Day 12	1.024	0.795	0.7761	24	19	120	0.9747	One-way ANOVA
	Day 0 vs Day 15	1.901	1.75	0.9202	24	11	120	0.4595	One-way ANOVA
	Day 0 vs Day 18	0.3885	-1.002	2.579	24	1	120	>0.9999	One-way ANOVA
	Day 0 vs Day 21	0.6897	0.8569	1.242	24	5	120	0.9988	One-way ANOVA
	Day 0 vs Day 24	1.187	-2.207	1.86	24	2	120	0.9338	One-way ANOVA
	Day 0 vs Day 27	0.3986	-0.6168	1.548	24	3	120	>0.9999	One-way ANOVA
	Day 0 vs Day 30	1.98	-2.46	1.242	24	5	120	0.4013	One-way ANOVA
Fig. S4a	(Day 15) Healthy1 Vehicle vs DMD1 Vehicle	4.395	-0.2387	0.05432	7	8	230	0.0005	One-way ANOVA
	(Day 15) Healthy1 Vehicle vs DMD1 4-OH Tam	1.849	-0.09382	0.05075	7	11	230	0.8701	One-way ANOVA
	(Day 15) DMD1 Vehicle vs DMD1 4-OH Tam	2.971	0.1449	0.04877	8	11	230	0.0939	One-way ANOVA
	(Day 18) Healthy1 Vehicle vs DMD1 Vehicle	1.612	-0.1544	0.09581	3	2	230	0.968	One-way ANOVA
	(Day 18) Healthy1 Vehicle vs DMD1 4-OH Tam	2.149	0.1504	0.06997	3	9	230	0.6307	One-way ANOVA
	(Day 18) DMD1 Vehicle vs DMD1 4-OH Tam	3.715	0.3048	0.08205	2	9	230	0.0076	One-way ANOVA
	(Day 21) Healthy1 Vehicle vs DMD1 Vehicle	1.919	-0.1685	0.08781	2	5	230	0.824	One-way ANOVA

Panel	Comparison	t	Mean Difference	SE of Mean Diff	n1	n2	df	P value	Test
Fig. S4a cont.	(Day 21) Healthy1 Vehicle vs DMD1 4-OH Tam	1.69	-0.1484	0.08781	2	5	230	0.9455	One-way ANOVA
	(Day 21) DMD1 Vehicle vs DMD1 4-OH Tam	0.3028	0.0201	0.06638	5	5	230	>0.9999	One-way ANOVA
	(Day 24) Healthy1 Vehicle vs DMD1 Vehicle	2.936	-0.1866	0.06355	5	6	230	0.1041	One-way ANOVA
	(Day 24) Healthy1 Vehicle vs DMD1 4-OH Tam	2.314	-0.2032	0.08781	5	2	230	0.48	One-way ANOVA
	(Day 24) DMD1 Vehicle vs DMD1 4-OH Tam	0.1933	-0.01656	0.0857	6	2	230	>0.9999	One-way ANOVA
	(Day 27) Healthy1 Vehicle vs DMD1 Vehicle	1.747	-0.1265	0.07243	7	3	230	0.9233	One-way ANOVA
	(Day 27) Healthy1 Vehicle vs DMD1 4-OH Tam	1.171	0.08484	0.07243	7	3	230	0.9998	One-way ANOVA
	(Day 27) DMD1 Vehicle vs DMD1 4-OH Tam	2.466	0.2113	0.0857	3	3	230	0.3526	One-way ANOVA
	(Day 30) Healthy1 Vehicle vs DMD1 Vehicle	1.578	-0.09442	0.05983	8	5	230	0.9752	One-way ANOVA
	(Day 30) Healthy1 Vehicle vs DMD1 4-OH Tam	0.6014	0.03598	0.05983	8	5	230	>0.9999	One-way ANOVA
	(Day 30) DMD1 Vehicle vs DMD1 4-OH Tam	1.965	0.1304	0.06638	5	5	230	0.7898	One-way ANOVA
Fig. S4b	(Day 3) Healthy1 Vehicle vs DMD1 Vehicle	0.07407	-0.01804	0.2435	10	10	228	>0.9999	One-way ANOVA
	(Day 3) Healthy1 Vehicle vs DMD1 4-OH Tam	0.6406	0.134	0.2092	10	21	228	>0.9999	One-way ANOVA
	(Day 3) DMD1 Vehicle vs DMD1 4-OH Tam	0.7268	0.152	0.2092	10	21	228	>0.9999	One-way ANOVA
	(Day 6) Healthy1 Vehicle vs DMD1 Vehicle	1.559	0.3464	0.2223	12	12	228	0.9787	One-way ANOVA
	(Day 6) Healthy1 Vehicle vs DMD1 4-OH Tam	2.913	0.5691	0.1954	12	22	228	0.1116	One-way ANOVA
	(Day 6) DMD1 Vehicle vs DMD1 4-OH Tam	1.14	0.2227	0.1954	12	22	228	0.9999	One-way ANOVA
	(Day 9) Healthy1 Vehicle vs DMD1 Vehicle	0.8646	-0.2163	0.2502	10	9	228	>0.9999	One-way ANOVA
	(Day 9) Healthy1 Vehicle vs DMD1 4-OH Tam	0.489	0.104	0.2127	10	19	228	>0.9999	One-way ANOVA

Panel	Comparison	t	Mean Difference	SE of Mean Diff	n1	n2	df	P value	Test
Fig. S4b cont.	(Day 9) DMD1 Vehicle vs DMD1 4-OH Tam	1.454	0.3203	0.2203	9	19	228	0.9916	One-way ANOVA
	(Day 12) Healthy1 Vehicle vs DMD1 Vehicle	1.849	0.4202	0.2273	11	12	228	0.87	One-way ANOVA
	(Day 12) Healthy1 Vehicle vs DMD1 4-OH Tam	0.4987	0.1029	0.2063	11	19	228	>0.9999	One-way ANOVA
	(Day 12) DMD1 Vehicle vs DMD1 4-OH Tam	1.581	-0.3173	0.2008	12	19	228	0.9747	One-way ANOVA
	(Day 15) Healthy1 Vehicle vs DMD1 Vehicle	0.0082	0.0022	0.2722	8	8	228	>0.9999	One-way ANOVA
	(Day 15) Healthy1 Vehicle vs DMD1 4-OH Tam	1.811	0.4581	0.253	8	11	228	0.892	One-way ANOVA
	(Day 15) DMD1 Vehicle vs DMD1 4-OH Tam	1.802	0.4558	0.253	8	11	228	0.8968	One-way ANOVA
	(Day 18) Healthy1 Vehicle vs DMD1 Vehicle	1.345	0.6342	0.4715	4	2	228	0.9974	One-way ANOVA
	(Day 18) Healthy1 Vehicle vs DMD1 4-OH Tam	1.432	0.8718	0.6087	4	1	228	0.9933	One-way ANOVA
	(Day 18) DMD1 Vehicle vs DMD1 4-OH Tam	0.3563	0.2376	0.6668	2	1	228	>0.9999	One-way ANOVA
	(Day 21) Healthy1 Vehicle vs DMD1 Vehicle	1.292	0.5885	0.4555	2	5	228	0.9986	One-way ANOVA
	(Day 21) Healthy1 Vehicle vs DMD1 4-OH Tam	1.471	0.6701	0.4555	2	5	228	0.9901	One-way ANOVA
	(Day 21) DMD1 Vehicle vs DMD1 4-OH Tam	0.237	0.0816	0.3443	5	5	228	>0.9999	One-way ANOVA
	(Day 24) Healthy1 Vehicle vs DMD1 Vehicle	0.699	0.2305	0.3297	5	6	228	>0.9999	One-way ANOVA
	(Day 24) Healthy1 Vehicle vs DMD1 4-OH Tam	0.2539	0.1157	0.4555	5	2	228	>0.9999	One-way ANOVA
	(Day 24) DMD1 Vehicle vs DMD1 4-OH Tam	0.2582	-0.1148	0.4445	6	2	228	>0.9999	One-way ANOVA
	(Day 27) Healthy1 Vehicle vs DMD1 Vehicle	0.1148	-0.04315	0.3757	7	3	228	>0.9999	One-way ANOVA
	(Day 27) Healthy1 Vehicle vs DMD1 4-OH Tam	0.6766	-0.2542	0.3757	7	3	228	>0.9999	One-way ANOVA
	(Day 27) DMD1 Vehicle vs DMD1 4-OH Tam	0.4748	-0.2111	0.4445	3	3	228	>0.9999	One-way ANOVA

Panel	Comparison	t	Mean Difference	SE of Mean Diff	n1	n2	df	P value	Test
Fig. S4b cont.	(Day 30) Healthy1 Vehicle vs DMD1 Vehicle	0.2624	0.07969	0.3037	9	5	228	>0.9999	One-way ANOVA
	(Day 30) Healthy1 Vehicle vs DMD1 4-OH Tam	0.587	0.1783	0.3037	9	5	228	>0.9999	One-way ANOVA
	(Day 30) DMD1 Vehicle vs DMD1 4-OH Tam	0.2863	0.09858	0.3443	5	5	228	>0.9999	One-way ANOVA
Fig. S5a Healthy1 Vehicle	Day 0 vs Day 3	2.897	-39.81	13.74	23	13	69	0.0493	One-way ANOVA
	Day 0 vs Day 6	2.213	-32.13	14.52	23	11	69	0.264	One-way ANOVA
	Day 0 vs Day 9	0.5123	-8.758	17.1	23	7	69	>0.9999	One-way ANOVA
	Day 0 vs Day 12	0.1613	-2.758	17.1	23	7	69	>0.9999	One-way ANOVA
	Day 0 vs Day 15	0.7913	13.53	17.1	23	7	69	0.9965	One-way ANOVA
	Day 0 vs Day 18	0.7449	14.56	19.54	23	5	69	0.9978	One-way ANOVA
	Day 0 vs Day 21	0.04292	-1.043	24.31	23	3	69	>0.9999	One-way ANOVA
	Day 0 vs Day 24	0.6835	19.96	29.2	23	2	69	0.999	One-way ANOVA
	Day 0 vs Day 27	0.1225	4.957	40.46	23	1	69	>0.9999	One-way ANOVA
	Day 0 vs Day 30	0.1225	4.957	40.46	23	1	69	>0.9999	One-way ANOVA
Fig. S5a DMD1 Vehicle	Day 0 vs Day 3	3.974	-41.72	10.5	26	18	55	0.0015	One-way ANOVA
	Day 0 vs Day 6	2.23	-28.41	12.74	26	10	55	0.1827	One-way ANOVA
	Day 0 vs Day 9	1.594	-29.31	18.38	26	4	55	0.5559	One-way ANOVA
	Day 0 vs Day 12	1.167	-29.31	25.12	26	2	55	0.8459	One-way ANOVA
	Day 0 vs Day 15	0.4102	-14.31	34.88	26	1	55	0.9995	One-way ANOVA
	Day 0 vs Day 18	0.2382	-8.308	34.88	26	1	55	0.9997	One-way ANOVA
	Day 0 vs Day 21	0.2779	9.692	34.88	26	1	55	0.9997	One-way ANOVA
Fig. S5a DMD1 4-OH Tam	Day 0 vs Day 3	1.484	-10.67	7.192	38	27	97	0.6551	One-way ANOVA
	Day 0 vs Day 6	0.8691	7.105	8.176	38	18	97	0.9675	One-way ANOVA
	Day 0 vs Day 9	0.7588	7.705	10.16	38	10	97	0.9847	One-way ANOVA
	Day 0 vs Day 12	1.489	15.77	10.59	38	9	97	0.6513	One-way ANOVA
	Day 0 vs Day 15	0.9982	-28.89	28.95	38	1	97	0.9332	One-way ANOVA

Panel	Comparison	t	Mean Difference	SE of Mean Diff	n1	n2	df	P value	Test
Fig. S5a DMD1 4-OH Tam cont.	Day 0 vs Day 18	0.3764	-10.89	28.95	38	1	97	0.9998	One-way ANOVA
	Day 0 vs Day 21	0.03818	1.105	28.95	38	1	97	>0.9999	One-way ANOVA
Fig. S5b Healthy1 Vehicle cont.	Day 0 vs Day 3	1.387	0.3665	0.2642	23	13	69	0.8093	One-way ANOVA
	Day 0 vs Day 6	0.3867	0.1079	0.2791	23	11	69	0.9996	One-way ANOVA
	Day 0 vs Day 9	2.451	0.8057	0.3287	23	7	69	0.1453	One-way ANOVA
	Day 0 vs Day 12	0.2281	0.07498	0.3287	23	7	69	0.9997	One-way ANOVA
	Day 0 vs Day 15	0.7796	-0.2563	0.3287	23	7	69	0.9931	One-way ANOVA
	Day 0 vs Day 18	0.7128	0.2678	0.3757	23	5	69	0.9965	One-way ANOVA
	Day 0 vs Day 21	1.94	0.9068	0.4674	23	3	69	0.4077	One-way ANOVA
	Day 0 vs Day 24	0.1254	0.07041	0.5614	23	2	69	0.9999	One-way ANOVA
	Day 0 vs Day 27	1.415	1.101	0.7779	23	1	69	0.7917	One-way ANOVA
	Day 0 vs Day 30	1.366	1.062	0.7779	23	1	69	0.8224	One-way ANOVA
Fig. S5b DMD1 Vehicle	Day 0 vs Day 3	2.197	0.3651	0.1662	23	16	48	0.1987	One-way ANOVA
	Day 0 vs Day 6	2.234	0.4484	0.2007	23	9	48	0.184	One-way ANOVA
	Day 0 vs Day 9	0.9222	0.255	0.2766	23	4	48	0.9469	One-way ANOVA
	Day 0 vs Day 12	1.392	0.7258	0.5215	23	1	48	0.7033	One-way ANOVA
	Day 0 vs Day 15	0.5032	0.2624	0.5215	23	1	48	0.9977	One-way ANOVA
	Day 0 vs Day 18	0.8905	-0.4644	0.5215	23	1	48	0.9555	One-way ANOVA
	Day 0 vs Day 21	0.6464	0.3371	0.5215	23	1	48	0.9925	One-way ANOVA
Fig. S5b DMD1 4-OH Tam	Day 0 vs Day 3	1.077	-0.1981	0.184	36	21	84	0.8899	One-way ANOVA
	Day 0 vs Day 6	0.9963	-0.1965	0.1972	36	17	84	0.9231	One-way ANOVA
	Day 0 vs Day 9	3.157	-0.8268	0.2619	36	8	84	0.0152	One-way ANOVA
	Day 0 vs Day 12	0.00201	0.000558	0.2768	36	7	84	>0.9999	One-way ANOVA
	Day 0 vs Day 15	2.098	-1.425	0.6793	36	1	84	0.2322	One-way ANOVA
	Day 0 vs Day 18	0.01081	-0.00734	0.6793	36	1	84	>0.9999	One-way ANOVA
	Day 0 vs Day 21	1.195	-0.8121	0.6793	36	1	84	0.8285	One-way ANOVA

Panel	Comparison	t	Mean Difference	SE of Mean Diff	n1	n2	df	P value	Test
Fig. S6a Line 1	Healthy1 Vehicle vs. DMD1 Vehicle	0.7917	0.01826	0.02306	72	44	177	0.8144	One-way ANOVA
	Healthy1 Vehicle vs. DMD1 4-OH Tam	4.046	0.08377	0.0207	72	64	177	0.0002	One-way ANOVA
	DMD1 Vehicle vs DMD1 4-OH Tam	2.776	0.06552	0.0236	44	64	177	0.0182	One-way ANOVA
Fig. S6a Line 2	Healthy2 Vehicle vs. DMD2 Vehicle	0.4689	-0.01057	0.02254	55	57	152	0.9533	One-way ANOVA
	Healthy2 Vehicle vs. DMD2 4-OH Tam	0.472	0.01146	0.02428	55	43	152	0.9524	One-way ANOVA
	DMD2 Vehicle vs DMD2 4-OH Tam	0.9145	0.02203	0.02409	57	43	152	0.7402	One-way ANOVA
Fig. S6b Line 1	Healthy1 Vehicle vs. DMD1 Vehicle	1.227	0.0143	0.01165	78	45	164	0.5283	One-way ANOVA
	Healthy1 Vehicle vs. DMD1 4-OH Tam	2.33	0.02734	0.01173	78	44	164	0.0618	One-way ANOVA
	DMD1 Vehicle vs DMD1 4-OH Tam	0.9884	0.01304	0.0132	45	44	164	0.6917	One-way ANOVA
Fig. S6b Line 2	Healthy2 Vehicle vs. DMD2 Vehicle	0.2528	0.004253	0.01682	50	34	115	0.9921	One-way ANOVA
	Healthy2 Vehicle vs. DMD2 4-OH Tam	2.362	0.03974	0.01682	50	34	115	0.0584	One-way ANOVA
	DMD2 Vehicle vs DMD2 4-OH Tam	1.933	0.03549	0.01836	34	34	115	0.1579	One-way ANOVA
Fig. S6c Line 1	Healthy1 Vehicle vs. DMD1 Vehicle	1.902	0.01825	0.00959	99	65	225	0.1653	One-way ANOVA
	Healthy1 Vehicle vs. DMD1 4-OH Tam	2.351	0.02266	0.00964	99	64	225	0.0576	One-way ANOVA
	DMD1 Vehicle vs DMD1 4-OH Tam	0.417	0.004413	0.01058	65	64	225	0.9663	One-way ANOVA
Fig. S6c Line 2	Healthy2 Vehicle vs. DMD2 Vehicle	0.465	0.006217	0.01337	59	57	163	0.9543	One-way ANOVA
	Healthy2 Vehicle vs. DMD2 4-OH Tam	1.892	0.02618	0.01384	59	50	163	0.1702	One-way ANOVA
	DMD2 Vehicle vs DMD2 4-OH Tam	1.431	0.01996	0.01395	57	50	163	0.3952	One-way ANOVA

Supplementary Table 2. Log-rank (Mantel-Cox) test

Panel	Comparison	df	Bonferroni corrected P value	Test
Fig. 5b	Healthy1 Vehicle vs. DMD1 Vehicle	1	0.2205	Log-rank (Mantel-Cox)
	Healthy1 Vehicle vs. DMD1 4-OH Tam	1	2.5569	Log-rank (Mantel-Cox)
	DMD1 Vehicle vs. DMD1 4-OH Tam	1	0.0741	Log-rank (Mantel-Cox)

Supplementary Table 3. Chi-square tests

	df = 2		Condition		
			Healthy1 Vehicle	DMD1 Vehicle	DMD1 4-OH Tamoxifen
Fig. 6d Line 1	Regular	Count	59	23	36
		% within Condition	61.5%	36.5%	62.1%
		Adjusted Residual	1.87	-3.38	1.37
		Corrected P-value	0.1845	0.0022	0.5121
	Arrhythmic	Count	37	40	22
		% within Condition	38.5%	63.5%	37.9%
		Adjusted Residual	-1.87	3.38	-1.37
		Corrected P-value	0.1845	0.0022	0.5121
	Total	Count	96	63	58

	df = 2		Condition		
			Healthy2 Vehicle	DMD2 Vehicle	DMD2 4-OH Tamoxifen
Fig. 6d Line 2	Regular	Count	47	23	26
		% within Condition	79.7%	40.4%	52.0%
		Adjusted Residual	4.23	-3.30	-1.00
		Corrected P-value	0.0001	0.0029	0.9519
	Arrhythmic	Count	12	34	24
		% within Condition	20.3%	59.6%	48.0%
		Adjusted Residual	-4.23	3.30	1.00
		Corrected P-value	0.0001	0.0029	0.9519
	Total	Count	59	57	50
